# Reduced YTHDF2 inhibits PD-L1 expression by stabilizing m^6^A-containing SPOP mRNA in colorectal cancer

**DOI:** 10.1038/s41419-026-08615-2

**Published:** 2026-03-24

**Authors:** Xian Xu, Hao Chen, Rongjie Zhao, Jiansheng Xie, Hao Liu, Binbin Xie, Jun Lou, Haidong Wang, Xinkai Wu, Weidong Han, Hongming Pan, Jiaying Shen

**Affiliations:** 1 Department of Medical Oncology, Sir Run Run Shaw Hospital, Zhejiang University School of Medicine, Hangzhou, Zhejiang China; 2 Department of Pulmonary and Critical Care Medicine, Sir Run Run Shaw Hospital, Zhejiang University School of Medicine, Hangzhou, Zhejiang China; 3 Department of Colorectal Medical Oncology, Zhejiang Cancer Hospital, Hangzhou, Zhejiang China

**Keywords:** Colorectal cancer, Tumour immunology

## Abstract

Colorectal cancer (CRC) is one of the most frequently diagnosed malignant tumors. However, clear evidence explaining the regulatory mechanisms of programmed death ligand 1 (PD-L1) in CRC has been limited. To illustrate the function of YTH N^6^-methyladenosine (m^6^A) RNA binding protein F2 (YTHDF2), we conducted a comprehensive evaluation of expression profiling datasets from online databases and clinical samples. We used a subcutaneous immunodeficient mouse model to investigate the impact of YTHDF2 on CRC. Western blots, flow cytometry, PD-1/PD-L1 binding assay, and cell killing assay were used to assess the relationship between YTHDF2 and PD-L1. We used RNA sequencing, along with methylated RNA immunoprecipitation (MeRIP) and RNA binding protein immunoprecipitation (RIP) sequencing to analyze mRNA expression, m^6^A methylation levels, and YTHDF2 target transcripts. The m^6^A methylation locations of mRNAs were verified using sequence-based RNA adenosine methylation site predictor (SRAMP), MeRIP-qRT-PCR, RIP-qRT-PCR, and a dual-luciferase reporter system. YTHDF2 was upregulated in CRC tissues, and patients with higher YTHDF2 expression had a worse prognosis. The in vivo model showed that YTHDF2 promoted CRC growth, whereas in vitro experiments showed that inhibiting YTHDF2 expression did not affect cell proliferation, migration, or invasion. Mechanistically, interference with YTHDF2 reduced PD-L1 expression and the binding ability between PD-1 and PD-L1. The use of RNA-seq, MeRIP-seq, RIP-seq, and bioinformatics tools confirmed that the speckle type BTB/POZ protein (*SPOP*) mRNA was a YTHDF2 target and validated its m^6^A methylation sites. After YTHDF2 knockdown, *SPOP* mRNA stability increased, causing an increase in SPOP expression and a decrease in PD-L1 expression. This study demonstrated that YTHDF2 might upregulate PD-L1 expression by destabilizing m^6^A-containing *SPOP* mRNA and promote CRC development. The biological effect of the YTHDF2-SPOP-PD-L1 axis presented a promising target for CRC treatment and provided an approach to enhance the efficacy of anti-PD-1/PD-L1 therapy.

## Introduction

Worldwide, CRC is a common malignancy of the digestive tract, with an incidence and mortality ranking third among all malignant tumors [1], and a similar burden can be seen in China [2]. For patients who receive surgery, 30 ~ 40% experience recurrence and metastasis within 5 years [3]. Although chemotherapy, molecular targeted therapy, and immunotherapy have shown clinical success, advanced patients are still confronted with poor prognoses and low survival rates [4]. Research progress has revealed some mechanisms underlying the development and occurrence of CRC, such as the roles of the tumor microenvironment [5].

PD-L1, coded by *CD274*, is a crucial immune regulator that plays an essential biological role in subverting the anti-cancer activity of T cells [6]. Anti-PD-1/PD-L1 agents act on T cells and relieve immune suppression, making them the most promising checkpoint inhibitors. Regarding CRC, anti-PD-1 agents elicit significant clinical responses in microsatellite instability-high (MSI-H)/deficient mismatch repair (dMMR) patients [7]. However, anti-PD-1 agents are unable to overcome primary drug resistance in approximately 30% of CRC patients, and they are mainly ineffective in microsatellite stability (MSS) and mismatch repair-proficient (pMMR) CRC patients [8]. Thus, it is especially meaningful to explore the mechanisms and effects of the PD-1/PD-L1 response in CRC.

With advancements in technology, various forms of RNA modifications have been discovered. As the most prevalent RNA modification, m^6^A is extensively studied for its vital roles in various biological processes, such as RNA processing, translation, and degradation [9]. Moreover, m^6^A regulators are reported to participate in immune responses, including the regulation of macrophages [10], dendritic cells [11], natural killer cells [12], and T cells [13]. m^6^A regulators that exert dynamic regulatory effects are classified into writers, erasers, and readers. YTHDF2 is a reader that primarily recognizes and binds to m^6^A-modified sites on mRNAs [14]. Studies have shown that YTHDF2 primarily participates in mRNA decay [15]. Moreover, YTHDF2 is found to be involved in immune responses and improvements in therapeutic efficacy [16, 17]. However, the impact of YTHDF2 on CRC remains unclear.

In our study, we proposed a regulatory relation between YTHDF2 and PD-L1 expression and explored the role of YTHDF2 in CRC progression, providing potential molecular markers and therapeutic targets for CRC patients.

## Materials and Methods

### Bioinformatics analysis

The RNA-seq data were obtained from The Cancer Genome Atlas (TCGA, 620 CRC tissues and 51 normal tissues) data portal (https://portal.gdc.cancer.gov/) and Genotype-Tissue Expression (GTEx, 779 normal tissues) database (https://www.gtexportal.org/home/index.html). Prognosis and expression information were provided by GSE17537 (*n* = 55), GSE31595 (*n* = 37), and GSE38832 (*n* = 122). We extracted pathological and molecular characteristics as well as expression data from GSE39582 (*n* = 585), and immunotherapy response information from IMvigor210 (*n* = 348) [18]. We collected *SPOP* mutation data from the Pan-Cancer Atlas project [19].

Analyses were performed in R software (version 4.2.2). Ggplot2 and pheatmap were used to depict the expression patterns of 19 m^6^A regulators [20–22] and multi-gene correlations [23, 24]. The log-rank test was performed in the Kaplan–Meier analysis. Differentially expressed genes were identified using the limma package. The gene set “c5.all.v7.1.symbols” was downloaded from the Gene-Set Enrichment Analysis (GSEA) database (https://www.gsea-msigdb.org) for gene ontology (GO) and GSEA enrichment analysis using the fgsea package. Consensus molecular subtypes (CMS) classification was defined using the CMSclassifier package [25]. The statistical difference between two groups was assessed with the Wilcox test, and the significance of the difference among three groups was assessed with the Kruskal-Wallis test. Spearman’s correlation was used to assess the association between non-normally distributed quantitative variables.

### Human sample collection

73 adjacent normal and 98 CRC tissues were collected from the National Human Genetic Resources Sharing Service Platform (Shanghai, P. R. China). Patients were not subjected to any pre-operative anti-cancer treatment. All CRC diagnoses were histologically confirmed, and all participants provided informed consent to participate in the study and to provide the study specimens. We obtained ethical approval from the Ethics Committee of Shanghai Outdo Biotech Company (YB M-05-02).

### Histology and immunohistochemical staining

Formalin-fixed (G1101, Servicebio, Hubei, P. R. China) and paraffin-embedded samples were sectioned at 5 μm and stained with hematoxylin and eosin (G1005, Servicebio, Hubei, P. R. China). Immunohistochemistry (IHC) was performed on samples using YTHDF2 antibody (ab246514, Abcam, Cambridge, MA, UK), PD-L1 antibody (13684, CST, BOS, USA), or CD8 (ab235951, Abcam, Cambridge, MA, UK). The IHC score was generated by assigning sub-scores for distribution (0-4) and intensity (0-3), which were multiplied to yield the immunoreactivity score. The percentage positivity was scored as 0 (no staining), 1 (1–25%), 2 (26–50%), 3 (51–75%), or 4 (76–100%). The staining intensity was scored as 0 (no staining), 1 (weak staining), 2 (moderate staining), or 3 (strong staining). The total IHC score was calculated as the sum of the above factors, which ranged from 0 to 12. The CD8^+^ T cell infiltration in tumor tissues was evaluated by staining with CD8, and quantification was performed using ImageJ software.

### Cell culture and reagents

Human CRC cell lines (RKO, LoVo, and HCT-116) and HEK-293T were purchased from the National Collection of Authenticated Cell Cultures (Shanghai, P. R. China). Cell identity was confirmed by short tandem repeat (STR) profiling. To exclude mycoplasma contamination, all cell lines were routinely tested using the MycoBlue Mycoplasma Detector (D101-01, Vazyme, Jiangsu, P. R. China) prior to experiments, and only mycoplasma-negative cells were used in this study. Cell lines were maintained in RPMI-1640 (MA0215, Meilunbio, Liaoning, P. R. China) or DMEM medium (MA0212, Meilunbio, Liaoning, P. R. China). All media were supplemented with 10% fetal bovine serum (FBS, NFBS-2500, Noverse^TM^, SN, Germany) and 1% penicillin/streptomycin (MA0110, Meilunbio, Liaoning, P. R. China). All cells were cultured at 37 °C in a humidified atmosphere containing 5% CO_2_. Cells in the logarithmic growth phase were used in the subsequent experiments.

The following reagents were used: MG132 (C2211, Sigma, MO, USA), cycloheximide (CHX) (C7698, Sigma, MO, USA), and chloroquine (CQ) (C6628, Sigma, MO, USA).

### Lentiviral production and transduction

All short hairpin RNA (shRNA) targeting sequences were cloned into hU6-MCS-CBh-gcGFP-IRES-puromycin vector (Genechem, Shanghai, P R. China). To produce lentivirus, HEK-293T cells were transfected with the aforementioned shRNA vectors and packaging plasmids pLP1, pLP2, and pLP/VSVG at a 4:3:3:2 ratio. Transfection was performed using polyetherimide (PEI) reagent (#24765, Polysciences, PA, USA). The viral supernatant was collected 48 h after transfection, filtered through a 0.45 μm filter, and used to infect RKO cells with polybrene (H8761, Solarbio, Beijing, P. R. China). 1 ng/μL puromycin (MB2005, Meilunbio, Liaoning, P. R. China) was applied for the following selection. shRNA targeting sequences are listed in Table S1.

### Primary cell culture

Peripheral blood mononuclear cells (PBMCs) were isolated through density gradient centrifugation of fresh blood from healthy volunteers, using Human peripheral lymphocyte separation medium (MB0911, Meilunbio, Liaoning, P. R. China). Written informed consent was obtained from each volunteer. PBMC single-cell suspensions were cultured in RPMI-1640 medium supplemented with ImmunoCult™ Human CD3/CD28 T Cell Activator (10971, Stemcell, VAN, Canada) and 10 ng/mL rIL-2 (589102, Biolegend, CA, USA). PBMC culturing was used to induce the activation and proliferation of T lymphocytes. All experimental procedures were approved by the Ethics Committee of Sir Run Run Shaw Hospital, Zhejiang University School of Medicine (SRRSH 2022-499-01).

### Animal experiments

A humanized mouse model was used to explore interactions between human immune and tumor cells. Non-obese diabetic, severe combined immunodeficiency gamma (NSG) immunodeficient mice were randomly allocated to the indicated treatment groups using a random number generator. 1 × 10^6^ RKO cells (sh-NC and sh-YTHDF2) were resuspended in 100 μL phosphate-buffered saline (PBS, CR20012, Cienry, Zhejiang, P. R. China) and injected subcutaneously into the dorsal right flank of 5-week-old male NSG mice (*n* = 5) purchased from Shanghai Model Organisms Center (Shanghai, P. R. China). 7 days after inoculation, mice received intravenous injection of 3 × 10^6^ activated PBMCs. For the treatment model, anti-PD-L1 (A2004, Selleck, TX, USA) or control immune globulin G (IgG, A2051, Selleck, TX, USA) was administered intraperitoneally on day 7 (200 μg/mouse). Tumor diameter was measured every 3 days for 3 weeks. Tumor volume (mm^3^) was estimated by measuring the longest and shortest diameters of the tumor. Tumor volume = 1/2 length × width^2^. When tumors reached the size limit (2 cm), mice were sacrificed, and tumors were isolated and weighed. All experimental procedures were approved by the Ethics Committee of Zhejiang University (ZJU20230100).

### Flow cytometry assay

Cultured cells were harvested using trypsin (CR25200, Cienry, Zhejiang, P. R. China), washed twice with ice-cold PBS, and resuspended. Tumors were dissociated into single-cell suspensions using RPMI-1640 medium containing 2% FBS and 2 mg/mL type Ⅳ collagenase (C5138, Sigma, MO, USA) in a shaker at 200 revolutions per minute and 37 °C for 2 h. Afterwards, 100 μL of the cell suspension (1 × 10^6^ cells/mL) was stained with the indicated antibodies according to the manufacturer’s instructions. Tumor cells were stained with anti-PD-L1 antibody (329705, Biolegend, CA, USA). Lymphocytes were stained with anti-CD45 (304016, Biolegend, CA, USA), anti-CD3 (300305, 317335, Biolegend, CA, USA), anti-CD4 (317415, Biolegend, CA, USA), and anti-CD8 (344705, Biolegend, CA, USA) antibodies. All live/dead discrimination was performed using the Auqa stain kit (L34966, Invitrogen, CA, USA). After incubation for 30 min at room temperature in the dark, the stained cells were washed with PBS and analyzed by flow cytometry (Beckman Coulter, CA, USA).

### Small interfering RNA (siRNA) transfection

SiRNAs against target genes were designed and synthesized by Tsingke Biotechnology (Beijing, P. R. China) and listed in Table S1. Oligonucleotides were transfected into cells using Lipofectamine^®^ RNAiMAX Reagent (13778, Invitrogen, CA, USA) according to the manufacturer’s recommendations. Subsequent experiments were conducted 48 h after transfection.

### Cell counting kit 8 (CCK-8)

Cell viability was assessed using CCK-8 reagent (MA0218, Meilunbio, Liaoning, P. R. China) according to the manufacturer’s recommendations. Cells were seeded into 96-well plates at a density of 2 × 10^3^ cells/well 24 h post-transfection. At 0, 24, 48, and 72 h after seeding, CCK-8 solution was added to each well in an amount equal to 10% of the culture medium volume and incubated at 37 °C for 1 h. The absorbance at 450 nm was measured using a microplate absorbance reader (Biotek, VT, USA).

### Trans-well migration and invasion assays

Cell migration and invasion assays were performed using 24-well trans-well chambers (3422, Corning, NY, USA). Cells were harvested and suspended in serum-free medium at a density of 2.5 × 10^5^/mL. Subsequently, 100 μL of cell suspension was added to the upper chamber, while the lower chamber was filled with 600 μL of medium containing 10% FBS. In the invasion assay, the upper chamber was pre-coated with Matrigel Matrix (354230, Corning, NY, USA) and incubated at 37 °C for 1 h. After incubation for 18 h, cells that did not invade through the membrane were mechanically removed with a cotton swab. Next, 4% paraformaldehyde (G1102, Servicebio, Hubei, P. R. China) was used to fix the cells on the bottom surface of the membrane. Then, cells were stained using a crystal violet solution (MA0148, Meilunbio, Liaoning, P. R. China) and imaged using a digital microscope (Carl Zeiss Jena, Germany). The number of cells was counted in 5 randomly selected fields.

### Isolation and transmission electron microscope (TEM) verification of exosomes

Cells were cultured in exosome-free medium for 48 h before collection. Exosome-depleted FBS was obtained by overnight ultracentrifugation at 100 000 *g* (Beckman Coulter, CA, USA). The medium was collected and purified by a standard differential centrifugation protocol [26]. The pellet was then resuspended in PBS and stored at −80 °C. The concentration of exosomes was quantified using a bicinchoninic acid (BCA) assay kit (GK5013, Generay, Shanghai, P. R. China).

For TEM verification of purified exosomes, 15 μL of exosomes were dropped onto formvar-carbon-coated nickel grids. After staining with 2% uranyl acetate, grids were air-dried and visualized using TEM (Hitachi, Switzerland) at Biosci Company (Hubei, P. R. China).

### Western blots

Cells were collected and lysed using NP-40 lysis buffer (MA0156, Meilunbio, Liaoning, P. R. China). Lysate samples were separated on gels and transferred to polyvinylidene fluoride membranes. After blocked with 5% nonfat dry milk in tris-buffered saline with Tween-20 (TBST), membranes were incubated with primary antibodies at 4 °C overnight. Antibodies for YTHDF2 (ab220163, Abcam Cambridge, MA, UK), PD-L1 (13684, CST, BOS, USA), SPOP (16750, Proteintech, Hubei, P. R. China), ALIX (A25326, ABclonal, Hubei, P. R. China), CD63 (A19023, ABclonal, Hubei, P. R. China), LC3B (L7543, Sigma, MO, USA), p53 (sc-126, Santa cruz, CA, USA), c-MYC (D84C12, CST, BOS, USA), BRD2 (sc-130707, Santa cruz, CA, USA) and GAPDH (AF0343, Elabscience, Hubei, P. R. China) were used. The following day, membranes were washed in TBST to remove non-specific binding antibodies and incubated with horseradish peroxidase-conjugated secondary antibodies (SY0115 and SY0119, Elabscience, Hubei, P. R. China) at room temperature for 1 h. By exposing the membranes to the chemiluminescence substrate (MA0186, Meilunbio, Liaoning, P. R. China), protein bands were visualized by ChemiScope 3300 Mini (Clinx, P. R. China) and Amersham Imager 600 (GE, BOS, USA). GAPDH was used as the loading control, and the indicated protein bands were quantified using ImageJ software.

### Reverse transcription PCR and quantitative real-time PCR

Total RNA was extracted from cells using Trizol reagent (R401-01, Vazyme, Jiangsu, P. R. China) according to the manufacturer’s instructions. Subsequently, 1 μg of RNA was reverse-transcribed into cDNA using the 1st Strand cDNA Synthesis Kit (R312, Vazyme, Jiangsu, P. R. China). The target genes and the reference gene HPRT1 were quantified using UltraSYBR Mixture (CW0957, CWBIO, Jiangsu, P. R. China) in the Roche LightCycler (Roche, USA). The primers used in qRT-PCR are listed in Table S2. Quantification was performed using the 2^-ΔΔCt^ formula, and the fold change (FC) of target genes was normalized by the internal control.

### PD-1/PD-L1 binding assay

Cells were cultured on coverslips, transfected with corresponding siRNAs, and fixed with 4% paraformaldehyde (G1102, Servicebio, Hubei, P. R. China) for 10 min. After blocked with 5% bovine serum albumin (A8010, Solarbio, Beijing, P. R. China) in PBST for 30 min, coverslips were incubated with recombinant human PD-1 FC chimera protein (1086-PD, R&D systems, MN, USA) at 4 °C overnight and then incubated with fluorescent conjugated secondary antibody (A-11013, Invitrogen, CA, USA) for 1 h at room temperature. Slides were imaged on a confocal microscope (Nikon, Tokyo, Japan) and captured with 5 randomly selected fields.

### Cell killing assay

CRC cells were plated in a 96-well plate (RKO 5 × 10^3^/well, LoVo 2 × 10^3^/well). Activated PBMCs were added at a 1:10 ratio (CRC cells/PBMCs) and incubated at 37 °C for 12 h. Caspase 3/7 substrate (22796, AAT Bioquest, CA, USA) was added to the plate and incubated for 1 h at room temperature according to the manufacturer’s instructions. Green-fluorescent cells were counted as dead cells. The fluorescence intensity (Ex/Em = 490/525 nm) was measured and normalized to CRC cells incubated in the absence of PBMCs to determine the percentage of dead cells.

### RNA-seq, MeRIP-seq, and RIP-seq

The immunoprecipitation (IP) protocols for MeRIP, RIP, and the RNA sequencing protocols in sh-NC and sh-YTHDF2 RKO cells were described previously [27] with the help of Lc-Bio Technologies (Hangzhou, P. R. China). Visualizations of m^6^A modifications and YTHDF2-binding sites on transcripts were generated using the integrative genomics viewer (IGV). Two replicates were used. The possible m^6^A methylation locations of mRNAs were verified using SRAMP [28].

### MeRIP-qPCR and RIP-qPCR

MeRIP-qPCR was performed using a Magna MeRIP^TM^ m^6^A kit (17-10499, Millipore, MA, USA) and a miRNeasy Mini Kit (217004, Qiagen, Hilden, Germany) according to the manufacturer’s protocols. Moreover, RIP was performed using a Magna RIP^TM^ kit (17-700, Millipore, MA, USA). An antibody targeting YTHDF2 (ab246514, Abcam, Cambridge, MA, UK) was applied to immunoprecipitate the mRNA-YTHDF2 complex. qRT-PCR was carried out following immunoprecipitation to quantify the changes in the m^6^A methylation level and YTHDF2-binding ability of target RNAs. The primers used are listed in Table S2.

### Dot blot assay

Total RNA was isolated with Trizol and denatured by heating at 95 °C for 3 min. Then, the RNA was cooled on ice. Then, 2 μL of RNA was spotted onto Nylon Transfer Membranes (YA1760, Solarbio, Beijing, P. R. China) and cross-linked using a UVP (BD, USA) at a total energy of 0.24 J/cm^2^. After overnight incubation at 4 °C with an anti-m^6^A antibody (ab284130, Abcam, Cambridge, MA, UK), the membranes were washed in TBST the next day and incubated with horseradish peroxidase-conjugated secondary antibodies at room temperature for 1 h. By exposing the membranes to the chemiluminescence substrate (MA0186, Meilunbio, Liaoning, P. R. China), m^6^A-modified dots were visualized by Amersham Imager 600 (GE, BOS, USA). Methylene blue was used as the loading control.

### Measurement of RNA lifespan

Actinomycin D (ActD, HY17559, MCE, NJ, USA) was added to CRC cells to inhibit mRNA transcription. Samples were harvested at 0, 3, and 6 h after ActD treatment. Total RNA was isolated and analyzed by qRT-PCR.

### Dual-luciferase reporter assay

The m^6^A sites were inserted into the pmirGLO vector (E1330, Promega, WI, USA), which was designed to study their effect on transcript stability. SPOP 3’ untranslated region (3’UTR) was cloned into the XhoI site of pmriGLO vector using ClonExpress® II One Step Cloning Kit (C112, Vazyme, Jiangsu, P. R. China). Both wild-type (WT) and mutant-type (Mut) m^6^A sequences in SPOP 3’UTR are listed in Table S3. Primers used for SPOP 3’UTR amplification and mutation are listed in Table S4. All constructs were confirmed by DNA sequencing. Transfection of the vector was performed with Lipofectamine 3000 reagent (L3000015, Invitrogen, CA, USA) according to the manufacturer’s instructions. A dual-Luciferase Assay kit (RG027, Beyotime, Shanghai, P. R. China) was used to assess mRNA expression in cells 48 h after transfection.

### Statistical analysis

All data were presented as mean ± standard error of mean (SEM). The majority of experiments were repeated two or three times. The hypothesis test for significance between two groups used the two-sided Student’s *t*-test. For three or more groups, results were analyzed with one-way analysis of variance (ANOVA). The Chi-square test was used to analyze IHC results and clinical characteristics of CRC patients. All statistical analyses were conducted using GraphPad Prism or SPSS, and statistical significance was inferred at *P* ≤ 0.050.

## Results

### YTHDF2 was a pro-tumorigenic factor in CRC and was closely correlated with immune-related pathways

**Fig. 1:**
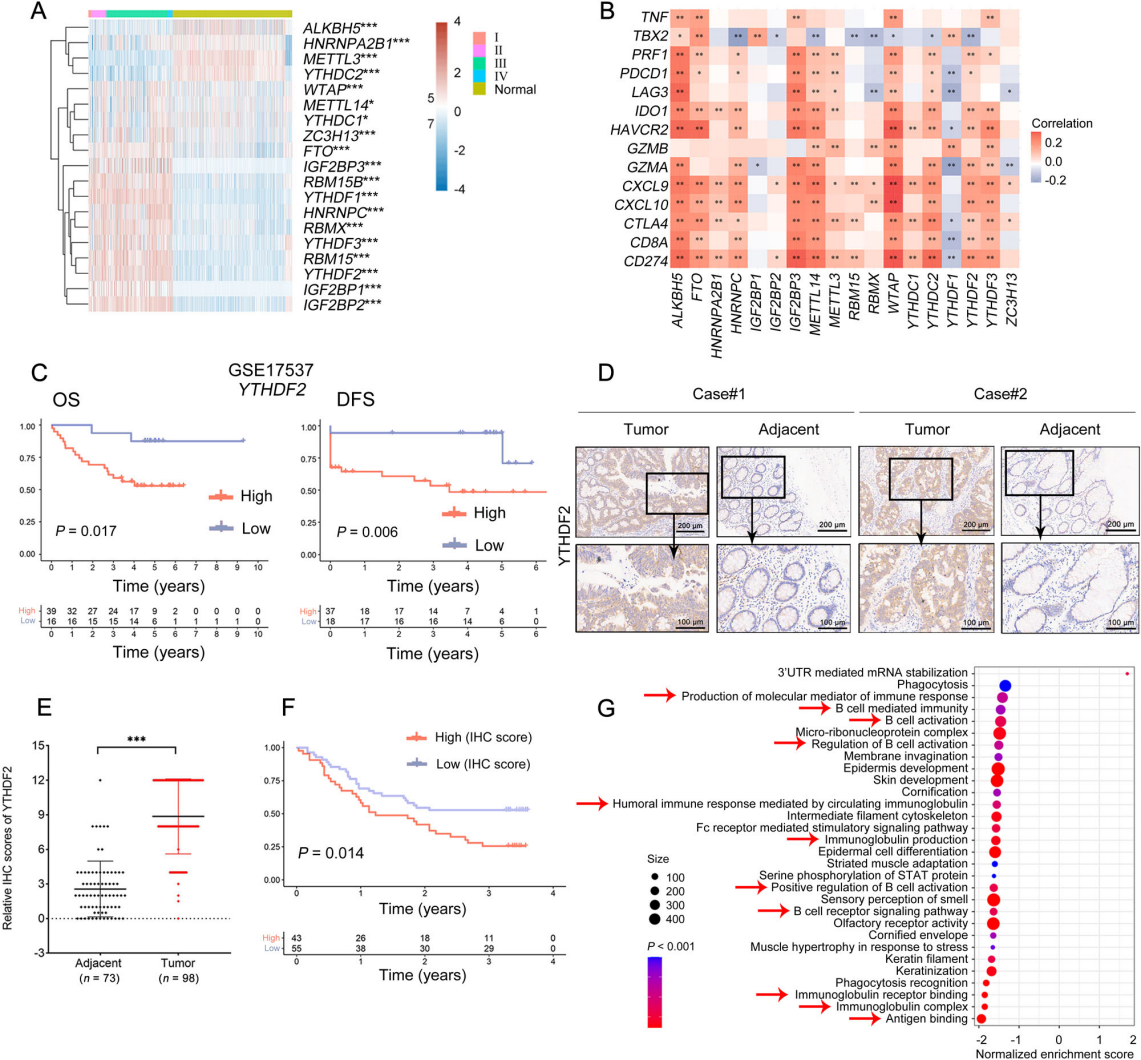
YTHDF2 was a pro-tumorigenic factor in CRC and was closely correlated with immune-related pathways. **A** Heatmap showed the expression pattern of 19 m^6^A regulators in 620 CRC tissues from the TCGA database, and 830 normal tissues from the TCGA database (*n* = 51) and the GTEx database (*n* = 779). The abscissa with different colors represented different groups, and the ordinate represented m^6^A regulators. Ⅰ-Ⅳ was related to tumor stage. The “writers” included METTL3, METTL14, WTAP, RBM15, RBM15B, and ZC3H13. The “readers” comprised YTHDC1, YTHDC2, YTHDF1, YTHDF2, YTHDF3, RBMX, HNRNPC, HNRNPA2B1, IGF2BP1, IGF2BP2, and IGF2BP3. The “erasers” included ALKBH5 and FTO. **B** Heatmap of the correlation among m^6^A regulators and immune-related genes in 620 CRC tissues from TCGA database. The abscissa and ordinate represented genes, different colors represented different correlation coefficients (red represented positive correlation whereas blue represented negative correlation). The darker the color was, the stronger the relation was. **C** Kaplan-Meier analysis of OS (left) and DFS (right) for patients with high or low *YTHDF2* expression levels from GSE17537 (*n* = 55). The top half of the figure showed the survival curves, and the bottom half of the figure represented the number of patients at different points in time. Red represented high expression level whereas blue represented low expression level. **D**, **E** Tissue sections from CRC patients were immunohistochemically stained for YTHDF2. Representative adjacent (*n* = 73) and tumor (*n* = 98) tissues were shown. Scale bars, 200 μm and 100 μm. The quantification was shown (**E**). **F** Survival analysis for patients with high (IHC score = 12) or low (IHC score < 12) YTHDF2 expression levels from CRC patients’ samples. **G** GO analysis of 89 rectal tissues from the TCGA database showed significant enrichment in immune-related pathways with high or low *YTHDF2* expression levels. The abscissa represented enrichment score, and the ordinate represented pathways. The size of the circles represented the number of genes enriched, and the color depth of the circles represented the significance levels. Red arrows indicated immune-related pathways. The statistical difference of two groups was compared through the Wilcox test, significance difference of three groups was tested with the Kruskal-Wallis test. Spearman’s correlation analysis was used to describe the correlation between quantitative variables without a normal distribution. Asterisks (*) stands for significance levels, *P < 0.050, **P < 0.010, and ***P < 0.001. DFS disease-free survival, IHC immunohistochemistry, OS overall survival.

To uncover the relationship between m^6^A regulators and CRC development, we systematically evaluated the expression patterns of 19 m^6^A regulators [29] in human CRC and normal tissues (Fig. 1A). The expression levels of 15 m^6^A regulators (*P* < 0.050), including the “reader” *YTHDF2* (*P* < 0.001), were clearly higher in CRC tissues than in normal tissues. In contrast, the expression levels of 4 m^6^A regulators were suppressed in CRC tissues (*P* < 0.001). Apart from that, close connections were observed between the expression of 18 m^6^A regulators and 14 immune-related genes (*P* < 0.050, Fig. 1B), which might explain the mechanisms underlying the above expression patterns.

To assess the effects of m^6^A regulators on prognosis, we stratified patients into high- and low-expression groups and plotted survival curves (GSE17537, *n* = 55; Figs. 1C and S1). The overall survival (OS) and disease-free survival (DFS) probability for patients with lower *YTHDF2* expression levels were higher than for those with overexpressed *YTHDF2* (*P* < 0.050). In addition, other m^6^A regulators did not show a significant effect on prognosis (Fig. S1, *P* > 0.050).

To obtain further information on the clinicopathological features of YTHDF2, we collected 98 CRC patient tissues, 73 normal tissues, and corresponding clinical information. It was evident that the expression of YTHDF2 in CRC tissues was higher than that in normal tissues (Fig. 1D-E, Table 1, *P* < 0.001). Higher YTHDF2 expression was associated with older age (*P* = 0.016), higher histologic grade (*P* = 0.001), higher T stage (Table 2, *P* = 0.019), and worse prognosis (Fig. 1F, *P* = 0.014). We also conducted GO enrichment analysis and found that the differentially expressed genes were enriched in biological processes closely related to immunity (Fig. 1G, *P* < 0.001). Collectively, these results enabled us to understand how m^6^A regulators, especially YTHDF2, might affect CRC development.

**Table 1:** Differential expression of YTHDF2 in colorectal cancer and normal tissues.

Tissue types	Total cases	YTHDF2 expression, cases (%)		χ2	*P* value
		High	Low		
Colorectal cancer	98	43 (43.88)	55 (56.12)	39.56	<0.001
Adjacent tissues	73	1 (1.37)	72 (98.63)

**Table 2:** Correlation between YTHDF2 expression and clinicopathological characteristics.

Variables		Total	YTHDF2 expression, cases (%)		χ2	*P* value
			Low	High		
Age (year)					5.797	0.016
	<70	42	29 (69.05)	13 (30.95)		
	≥70	50	22 (44.00)	28 (56.00)		
Gender					0.240	0.624
	Female	42	25 (59.52)	17 (40.48)		
	Male	55	30 (54.55)	25 (45.45)		
Grade					10.622	0.001
	1–2	71	47 (66.20)	24 (33.80)		
	3	27	8 (29.63)	19 (70.37)		
T stage					5.509	0.019
	T_1_-T_3_	82	50 (60.98)	32 (39.02)		
	T_4_	12	3 (25.00)	9 (75.00)		
N stage					0.031	0.861
	N_0_	61	35 (57.38)	26 (42.62)		
	N_1_-N_2_	36	20 (55.56)	16 (44.44)		
TNM stage					0.031	0.861
	I-II	61	35 (57.38)	26 (42.62)		
	III-IV	36	20 (55.56)	16 (44.44)		

### YTHDF2-induced CRC tumor progression was independent of the proliferation, migration, or invasion of cells

**Fig. 2:**
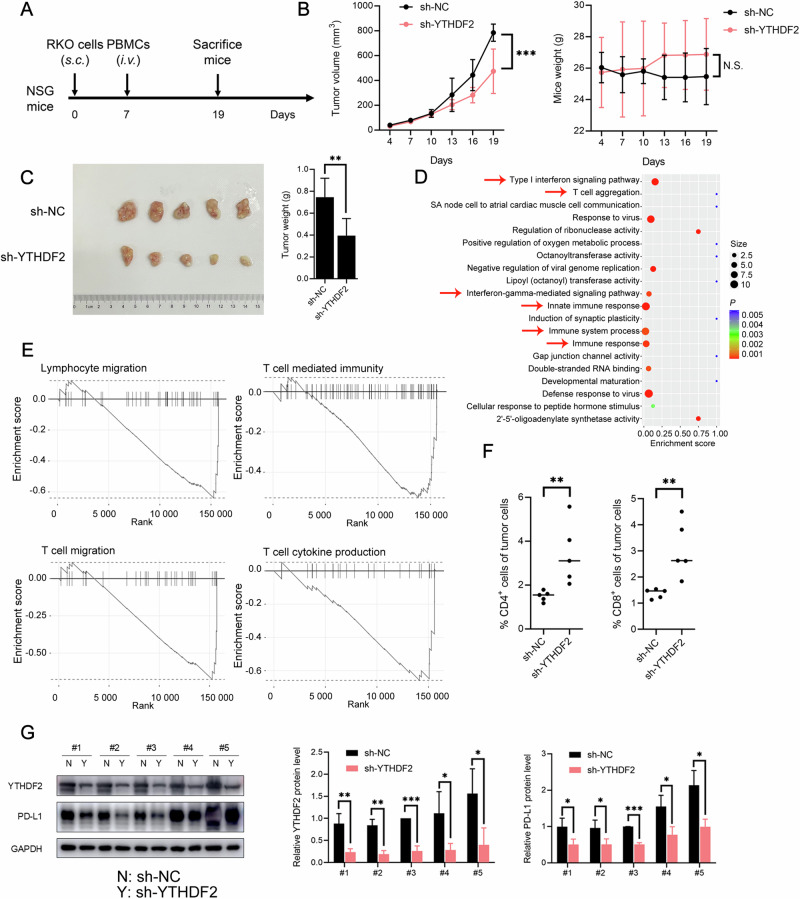
YTHDF2-induced tumor progression was independent of proliferation, migration, or invasion of cells. **A**, **B** Experimental design for the humanized mouse model (**A**). NSG mice were injected subcutaneously with 1 × 10^6^ sh-NC or sh-YTHDF2 RKO cells. 7 days after inoculation, mice received intravenous injection of 3 × 10^6^ activated PBMCs. The tumor volumes (left) and mice weights (right) were monitored after cell implanting every 3 days (*n* = 5) (**B**). When tumors reached the size limit (2 cm), mice were sacrificed. **C** Representative images of subcutaneous tumors formed by sh-NC or sh-YTHDF2 RKO cells (left). Tumor masses were weighed after harvesting (right). **D** GO analysis of significantly upregulated and down-regulated genes identified by RNA-seq data in sh-NC and sh-YTHDF2 RKO cells. The abscissa represented enrichment score, and the ordinate represented pathways. The size of the circles represented the number of genes enriched, and the color depth of the circles represented the significance levels. Red arrows indicated immune-related pathways. **E** GSEA showed the pathways of differentially expressed genes altered by YTHDF2. The abscissa represented genes arranged in order of fold change, and the ordinate represented enrichment score. **F** Flow cytometric analysis of the percentage of CD4^+^ (left) or CD8^+^ (right) tumor infiltrating-lymphocytes. **G** Western blots analysis of YTHDF2 and PD-L1 protein expression level in paired subcutaneous tumors (left). GAPDH was included as the loading control. The quantifications of YTHDF2 (middle) and PD-L1 (right) were shown. The hypothesis test for significance between two groups utilized the Student’s *t*-test. Results were analyzed with ANOVA for three or more groups. N.S., no statistical significance, *P < 0.050, **P < 0.010, and ***P < 0.001. Values are mean ± SEM. *i.v* intravenous injection, NC negative control, NSG non-obese diabetic, severe combined immunodeficiency gamma, PBMCs peripheral blood mononuclear cells, *s.c* subcutaneous injection.

To investigate the role of YTHDF2 in CRC progression, we successfully generated YTHDF2-knockdown RKO cells, labeled sh-NC and sh-YTHDF2 (Fig. S2A, B, P < 0.001). Using RNA-seq and GO analysis, these affected genes were found to be associated with immune-related pathways (Fig. 2D, *P* < 0.005). GSEA analysis (Fig. 2E) also showed that lymphocyte migration (Enrichment score = -0.639, *P* = 0.011), T-cell migration (Enrichment score = -0.677, *P* = 0.013), T-cell mediated immunity (Enrichment score = -0.527, *P* = 0.032), and T-cell cytokine production (Enrichment score = −0.658, *P* = 0.048) were correlated with *YTHDF2* expression. Based on these results, an immunodeficient mouse sub-skin model was applied (Fig. 2A). Human PBMCs were activated and amplified into T lymphocytes (Fig. S2C). The growth and weight of the subcutaneously transplanted tumors in the NSG mice decreased with YTHDF2 knockdown (Fig. 2B, *P* < 0.001; Fig. 2C, *P* < 0.010). Meanwhile, we observed no instances of lethal xenograft-versus-host disease, which could result in body weight loss (Fig. 2B, *P* > 0.050). Notably, we also established a mouse sub-skin model without PBMCs (Fig. S2D–F), in which the suppression of tumor growth caused by YTHDF2 knockdown disappeared (*P* > 0.050). We investigated tumor-infiltrating lymphocytes in subcutaneous tumors using flow cytometry and IHC. A higher count of CD4^+^ and CD8^+^ T cells (Fig. 2F, *P* < 0.010; Fig. S2F, *P* < 0.050) was observed. Additionally, our research confirmed that YTHDF2 was not associated with cell proliferation (Fig. S2G, S2I, P > 0.050), migration, or invasion (Fig. 2G, *P* > 0.050; Fig. S2H, S2J, *P* > 0.050).

Given the importance of PD-L1 in T-cell function, we used western blots to examine the relationship between YTHDF2 and PD-L1 in subcutaneous tumors (Fig. 2G). When YTHDF2 expression decreased, PD-L1 expression declined (*P* < 0.050; Fig. S2B, *P* < 0.050; Fig. S2K, *P* < 0.001). Besides, we explored the expression pattern of *YTHDF2* in patients after atezolizumab treatment (Fig. S3A). The results showed that patients with higher *YTHDF2* (*P* < 0.050) and *CD274* (*P* = 0.099) expression levels were more sensitive to atezolizumab treatment. Our data also showed that *YTHDF2* and *CD274* expression levels were significantly elevated in dMMR patients (Fig. S3B, *P* < 0.001) and in CMS1 patients [25, 30] (Fig. S3C, *P* < 0.001). In addition, we observed that *YTHDF2* expression was significantly increased in patients with Kirsten rat sarcoma viral oncogene (*KRAS*) and B-Raf proto-oncogene (*BRAF*) mutations (Fig. S3D, *P* < 0.010). Based on the above findings, we further investigated whether depleting YTHDF2 enhanced the response to immunotherapy in mouse models. NSG mice bearing tumors were treated with control IgG or anti-PD-L1 antibody. YTHDF2 knockdown synergized with anti-PD-L1 treatment, resulting in the inhibition of tumor growth and a decrease in tumor weight (Fig. S4A–D, *P* < 0.050). These effects were accompanied by increased CD4^+^ and CD8^+^ T cell infiltration (Fig. S4E, *P* < 0.050). Taken together, these results indicated that YTHDF2 was a key contributor to CRC progression, enabling tumor cells to increase PD-L1 expression and exhibit stronger resistance to T-cell-mediated cytotoxicity.

### Depletion of YTHDF2 decreased PD-L1 expression

**Fig. 3:**
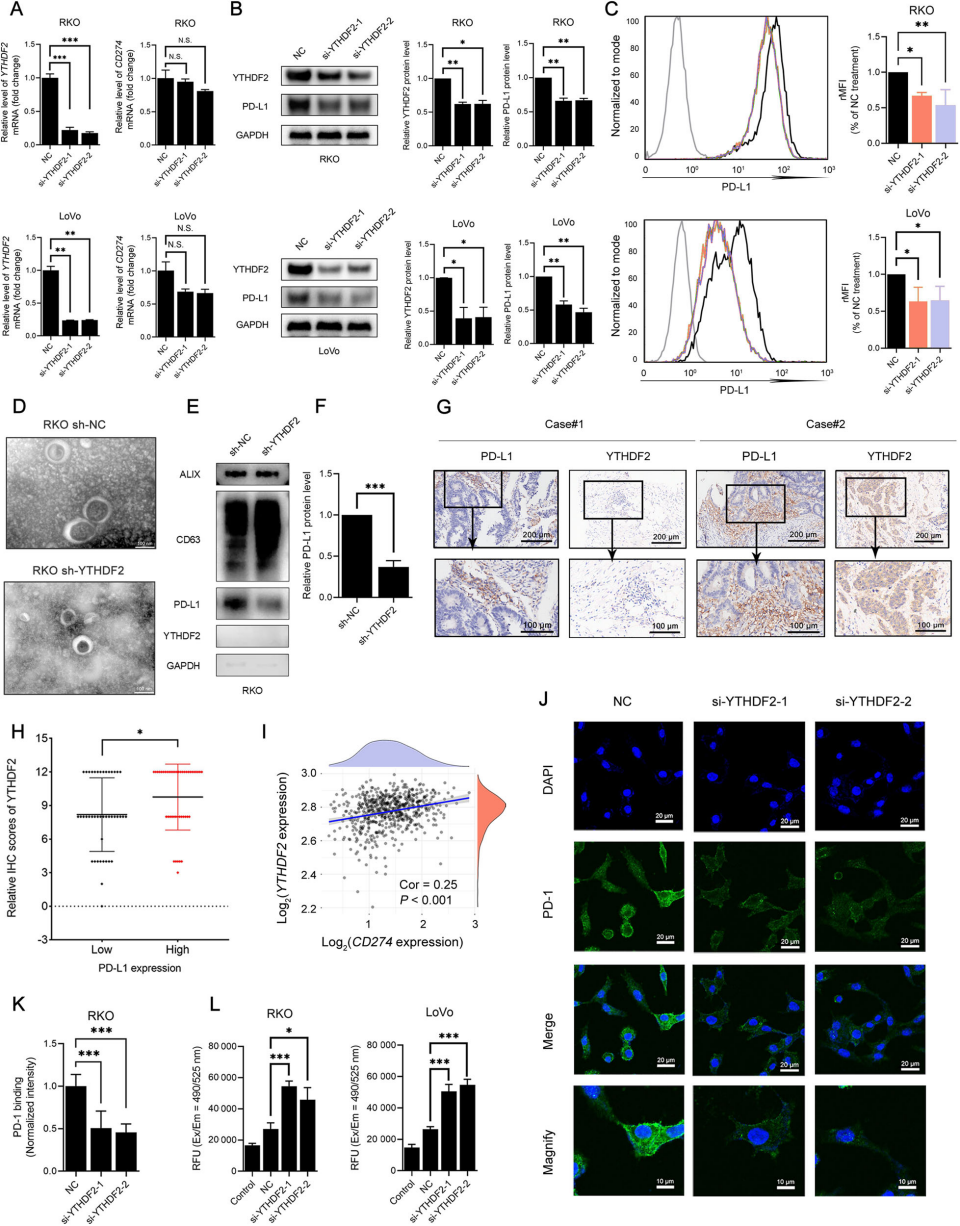
Depletion of YTHDF2 decreased PD-L1 expression. **A** qRT-PCR analysis of *YTHDF2* and *CD274* in siRNAs (NC or si-YTHDF2) transfected CRC cell lines (RKO, upper; LoVo, lower). HPRT1 served as the internal control. **B** Western blots (left) analysis of YTHDF2 and PD-L1 in siRNAs (NC or si-YTHDF2) transfected CRC cell lines (RKO, upper; LoVo, lower). GAPDH was included as the loading control. The quantifications of YTHDF2 (middle) and PD-L1 (right) were shown. **C** PD-L1 was visualized by flow cytometry (left) in RKO (upper) and LoVo (lower) cells treated with siRNAs (NC or si-YTHDF2). The quantification was shown (right). **D** Representative TEM images of purified exosomes from sh-NC and sh-YTHDF2 RKO cells. Scale bar, 100 nm. Western blots analysis of PD-L1 in the purified exosomes from sh-NC and sh-YTHDF2 RKO cells (**E**). The quantification was shown (**F**). CD63 was included as the loading control. **G**, **H** Tissue sections from 88 CRC patients were immunohistochemically stained for YTHDF2 and PD-L1. Representative IHC images were shown (**G**). Patients were divided into two groups based on median PD-L1 IHC score. Scale bars, 200 μm and 100 μm. The quantification was shown (**H**). **I** The correlation between *YTHDF2* and *CD274* in 620 CRC tissues from the TCGA database. The abscissa represented the expression distribution of *CD274*, and the ordinate represented the expression distribution of *YTHDF2*. The density curve on the right represented the trend in distribution of *YTHDF2*, the upper density curve represented the trend in distribution of *CD274*. The value on the bottom represented the correlation *P* value and correlation coefficient. **J**, **K** Immuno-stain of PD-1 (fused to Ig-Fc) on RKO cells treated by siRNAs (NC or si-YTHDF2) (**J**). The green fluorescence represented the binding between PD-1 and PD-L1. Scale bars, 20 μm (upper), 10 μm (magnified). The quantification was shown (**K**). **L** T cell killing assay of RKO (left) and LoVo (right) cells after YTHDF2 knockdown. The expression correlation of two genes was analyzed with Spearman. The hypothesis test for significance between two groups utilized the Student’s *t*-test. Results were analyzed with ANOVA for three or more groups. N.S., no statistical significance, *P < 0.050, **P < 0.010, and ***P < 0.001. Values are mean ± SEM. IHC immunohistochemistry, MFI mean fluorescence intensity, NC negative control, RFU relative fluorescence unit.

Based on the above results, it was reasonable to hypothesize that YTHDF2 could promote PD-L1-induced immunosuppression. After YTHDF2 knockdown, the protein expression level of PD-L1 decreased notably (Fig. 3B, *P* < 0.010; Fig. S5B, *P* < 0.050), whereas the mRNA expression level did not change (Fig. 3A, *P* > 0.050; Fig. S5A, *P* < 0.050) in RKO, LoVo, and HCT-116 cells. Accordingly, flow cytometry demonstrated that reduced YTHDF2 suppressed PD-L1 that resided on the surface membrane of CRC cells (Fig. 3C, *P* < 0.050; Fig. S5C, *P* < 0.010). Besides, previous studies indicated that exosomal PD-L1 also played an important role in anti-PD-1/PD-L1 immunotherapy [26, 31]. To investigate whether YTHDF2 affects PD-L1 levels in exosomes, we isolated exosomes from sh-NC and sh-YTHDF2 RKO cells and verified them by TEM (Fig. 3D). The exosomal markers CD63 and ALIX were also confirmed in the purified vesicles (Fig. 3E). Significantly, knockdown of YTHDF2 markedly reduced PD-L1 expression in exosomes (Fig. 3E, F, *P* < 0.001). Notably, GAPDH was detected only at very low levels in these exosomal fractions, whereas YTHDF2 was undetectable.

Additionally, 88 CRC tissues were analyzed and divided into high- and low-PD-L1 expression groups based on median IHC scores (Fig. 3G). Higher YTHDF2 expression was clearly more common in the group with higher PD-L1 expression (Fig. 3H, *P* < 0.050). We obtained similar results in 620 CRC tissues from the TCGA database (Fig. 3I, *P* < 0.001). The decreased levels of PD-L1, mediated by reduced YTHDF2, could attenuate the binding between PD-1 and PD-L1 (Fig. 3J, K, *P* < 0.001). This decrease in binding activity increased T-cell cytotoxicity (Fig. 3L, *P* < 0.050).

Clarifying the PD-L1 degradation pathway is crucial for elucidating the regulatory role of YTHDF2. Accordingly, we performed a CHX chase assay, which revealed a significant decrease in the half-life of PD-L1 protein upon YTHDF2 knockdown in RKO cells (Fig. S5D, *P* < 0.050), suggesting that YTHDF2 depletion compromised PD-L1 stability. Furthermore, treatment with the proteasome inhibitor MG132 effectively rescued the PD-L1 downregulation induced by YTHDF2 knockdown (Fig. S5E, F, *P* < 0.050). In contrast, the lysosomal inhibitor CQ showed no discernible effect on PD-L1 protein levels. Collectively, these results implied that YTHDF2 regulated PD-L1 primarily through the proteasome-dependent pathway rather than via lysosomal degradation.

Besides, knockdown of METTL3, METTL14, ALKBH5, YTHDF1, or YTHDF3 did not influence PD-L1 expression (Fig. S5G, H, *P* > 0.050). Also, knockdown of YTHDF2 did not impact the expression levels of METTL3, METTL14, ALKBH5, FTO, YTHDF1, or YTHDF3, suggesting that YTHDF2 regulated its mRNA targets independently (Fig. S6A-B, *P* > 0.050).

### Identification of potential target mRNAs of YTHDF2

Recent studies have established YTHDF2 as the m⁶A reader that primarily induces the decay of its target mRNAs [15], predicting a negative correlation with downstream gene expression. Our observation of a positive correlation between YTHDF2 and PD-L1 protein levels, therefore, pointed to an indirect regulatory mechanism. Then, we subjected sh-NC and sh-YTHDF2 RKO cells to RNA, MeRIP, and RIP sequencing to identify potential YTHDF2 targets.

**Fig. 4:**
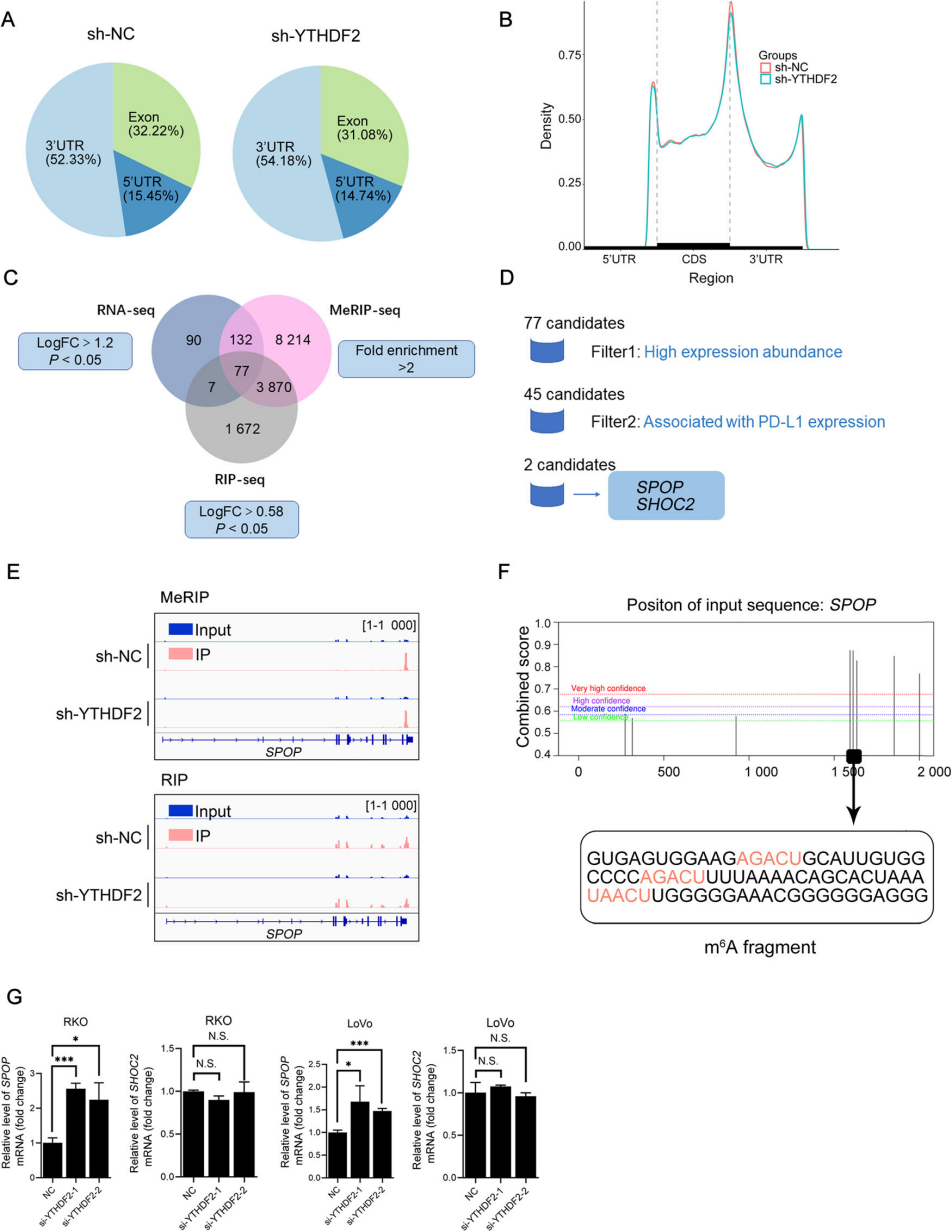
Identification of potential target mRNAs of YTHDF2. **A** Percentages of m^6^A modification sites in 5’UTR, exon, and 3’UTR. **B** Distribution of the enriched m^6^A peaks across mRNA transcripts and nearly unchanged distribution of m^6^A peaks between sh-NC and sh-YTHDF2 cells. **C** Venn diagram showed the overlap of transcripts identified by RNA-seq, MeRIP-seq, and RIP-seq. **D** Flow chart showed the selection for candidate YTHDF2 target transcripts. **E** IGV tracks displayed the distribution of m^6^A peaks and YTHDF2-binding sites along *SPOP* mRNAs according to MeRIP-seq (upper) and RIP-seq (lower) data. The abscissa represented transcripts, and the ordinate represented peak signals. Red represented the IP group, whereas blue represented the input group. **F** The potential m^6^A sites of *SPOP* mRNA were predicted by SRAMP. The abscissa represented transcripts, and the ordinate represented combined scores. Different color lines indicated different confidences (red, purple, blue, and green, respectively, represented very high, high, moderate, and low confidence). High score sites were marked by arrows. **G** qRT-PCR analysis of *SPOP* and *SHOC2* expression levels post siRNAs (NC or si-YTHDF2) transfection in CRC cell lines (RKO and LoVo). HPRT1 served as the internal control. The hypothesis test for significance between two groups utilized the Student’s *t*-test. Results were analyzed with ANOVA for three or more groups. N.S., no statistical significance, *P < 0.050, **P < 0.010, and ***P < 0.001. Values are mean ± SEM. 3’UTR: 3’ untranslated region, 5’UTR: 5’ untranslated region, CDS coding sequence, FC fold change, IP immunoprecipitation, m^6^A N^6^-methyladenosine, MeRIP methylated RNA immunoprecipitation, NC negative control, RIP RNA binding protein immunoprecipitation.

MeRIP-seq showed that m^6^A peaks mainly clustered in the 3’UTR around the stop codon (Fig. 4A, B), consistent with the distribution of m^6^A sites on mRNAs reported in previous studies (Fig. 4C, fold enrichment > 2) [32]. We observed 525 transcripts with increased levels and 534 transcripts with decreased levels (Fig. S7A, B). Among them, 306 transcripts were defined as significantly upregulated targets (Fig. 4C, logFC > 1.2, *P* < 0.050). In addition, we sequenced mRNAs obtained from RIP to identify YTHDF2-binding mRNAs (Fig. 4C, logFC > 0.58, *P* < 0.050). A “GGACU” motif was also identified in sh-NC and sh-YTHDF2 cells (Fig. S7C, *P* < 0.001). It was noteworthy that the previously identified m^6^A consensus motif “DRACH” (where D represents A, G, or U, R represents A or G, and H represents A, C, or U) was validated in our study [33]. Furthermore, YTHDF2-binding sites followed the same pattern, with a “GAACU” motif in sh-NC cells and an “AGACU” motif in sh-YTHDF2 cells (Fig. S7C, *P* < 0.001), which were consistent with the m^6^A consensus motif.

The initial screening for YTHDF2 target mRNAs was based on three criteria: upregulation, presence of m^6^A sites, and direct YTHDF2 binding (Fig. 4C). From this, 77 candidate transcripts were identified. After filtering out those with low expression, 45 candidates were retained for subsequent analysis (Fig. 4D). To further narrow down the list, we performed an extensive literature search to evaluate the likelihood of a functional association between each candidate and PD-L1, prioritizing those with evidence of interaction. Through this literature-based assessment, *SPOP* and SHOC2 leucine-rich repeat scaffold protein (*SHOC2*) stood out as the strongest candidates. Using IGV, we produced visualizations of m^6^A modifications and YTHDF2-binding sites on transcripts. Compared to the input, *SPOP* mRNA showed significant enrichment of both m^6^A modifications and YTHDF2 binding sites within its 3’UTR (Fig. 4E), indicating that YTHDF2 binding corresponded precisely to m^6^A-modified regions. In addition, SRAMP analysis predicted that m^6^A resided on the 1593, 1611, and 1633 adenine residues (Fig. 4F). Collectively, these data suggested that YTHDF2 likely recognized and bound to these specific m^6^A sites on the *SPOP* mRNA.

For *SHOC2*, a discrepancy was noted between the sequencing data, which showed co-localization of m^6^A modifications and YTHDF2 binding sites in the coding sequence (CDS, Fig. S7D), and the SRAMP prediction, which favored these sites in the 5’UTR region (Fig. S7F). This positional discordance diminished confidence in *SHOC2* as a primary target. Besides, a role for YTHDF2 in regulating *CD274* mRNA via direct binding was precluded by our data. *CD274* mRNA harbored m^6^A sites in its CDS and 3’UTR (Fig. S7E and S7G), but YTHDF2 binding levels showed no significant enrichment over the input control (Fig. S7E). Upon YTHDF2 knockdown, the *SPOP* mRNA expression level increased (Fig. 4G, *P* < 0.050), while the expression level of *SHOC2* remained unchanged (Fig. 4G, *P* > 0.050). Based on these integrated findings, we proposed a model wherein YTHDF2 recognized m^6^A motifs on *SPOP* mRNA, bound to these sites, and subsequently promoted its degradation.

### *SPOP* mRNA was a crucial target by which YTHDF2 regulated immune responses

**Fig. 5:**
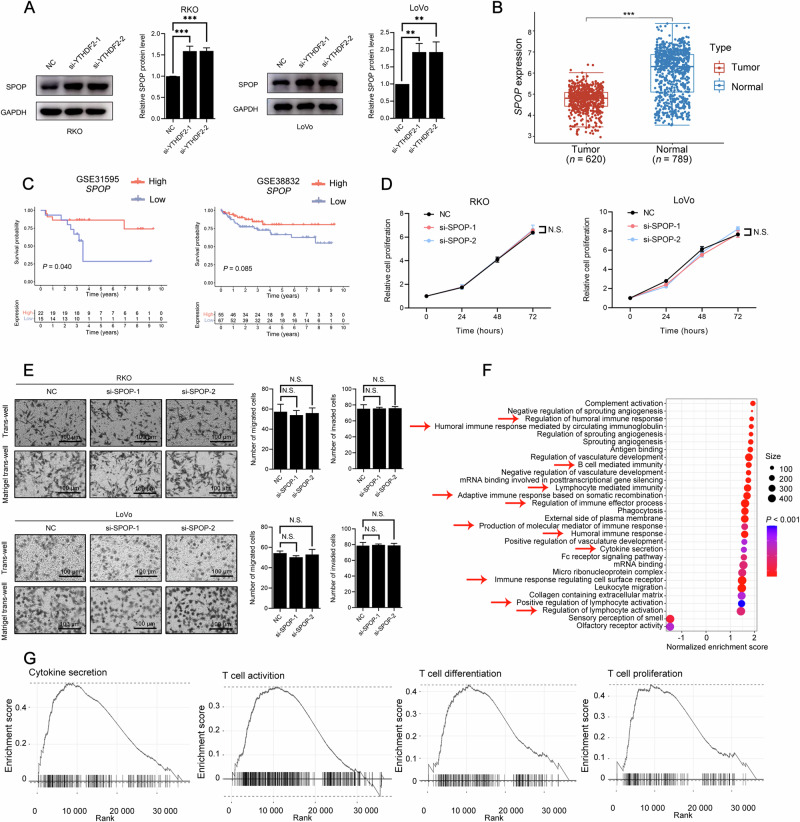
*SPOP* mRNA was a crucial target by which the YTHDF2 regulated immune responses. **A** Western blots analysis of SPOP expression levels post siRNAs (NC or si-YTHDF2) transfection in CRC cell lines (RKO, left; LoVo, right). **B** The expression distribution of *SPOP* in 620 CRC tissues and 789 normal tissues from TCGA and GTEx database. The abscissa represented groups, and the ordinate represented the expression distribution of SPOP. Different colors represented different groups. The statistical difference of two groups was compared through the Wilcox test. **C** Kaplan-Meier analysis for patients with high or low *SPOP* expression levels from GSE31595 (*n* = 37, left) and GSE38832 (*n* = 122, right). The top half of the figure showed the survival curves, and the bottom half of the figure represented the number of patients at different points of time. Red represented a high expression level, whereas blue represented a low expression level. **D** CCK-8 proliferation assay in siRNAs (NC or si-SPOP) transfected CRC cell lines (RKO, left; LoVo, right). **E** Representative images of trans-well migration and invasion assays in RKO cells (upper) and LoVo cells (lower) after SPOP knockdown (left). The quantification was shown (right). **F** GO analysis of 89 rectal cancer tissues from the TCGA database showed significant enrichment in the immune-related pathway between high and low *SPOP* expression (based on median expression) levels. The abscissa represented enrichment score, and the ordinate represented pathways. The size of the circles represented the number of genes enriched, and the color depth of the circles represented the significance levels. Red arrows indicated immune-related pathways. **G** GSEA showed the pathways of differentially expressed genes altered by *SPOP*. The abscissa represented genes arranged in order of fold change, and the ordinate represented enrichment score. The hypothesis test for significance between two groups utilized the Student’s *t*-test. Results were analyzed with ANOVA for three or more groups. GAPDH was included as the loading control in Western blots. HPRT1 served as the internal control in qRT-PCR. N.S., no statistical significance, *P < 0.050, **P < 0.010, and ***P < 0.001. Values are mean ± SEM. NC negative control.

As reported in previous studies, PD-L1 was regulated by the Cullin 3-SPOP E3 ligase via proteasome-mediated degradation [34, 35]. Based on these findings, we aimed to demonstrate the existence of the YTHDF2-SPOP-PD-L1 axis in CRC. After inhibition of YTHDF2, the expression of SPOP was obviously upregulated (Fig. 5A, *P* < 0.010; Fig. S7H, *P* < 0.010). Given the expression pattern and clinical significance of SPOP in CRC, it was unsurprising to find that *SPOP* was lower in tumor tissue (Fig. 5B, *P* < 0.001), whereas *YTHDF2* was upregulated (Fig. 1E). This inverse correlation in expression is in line with the proposed regulatory axis. Additionally, patients with elevated *SPOP* expression had better prognoses in both GSE31595 (Fig. 5C, *n* = 37, *P* = 0.040) and GSE38832 (*n* = 122, *P* = 0.085), indicating a tumor-suppressor role for *SPOP*. To further examine the effects of SPOP on CRC progression, cell proliferation, migration, and invasion assays were also performed. Our findings suggested that, consistent with YTHDF2, SPOP did not significantly affect any of these processes (Fig. 5D, E, *P* > 0.050).

To determine the possible function of SPOP, we classified patients into high- or low-*SPOP* expression groups. Through GO analysis, we discovered a correlation between *SPOP* and immune-related pathways (Fig. 5F, *P* < 0.001). With the help of GSEA (Fig. 5G), we also found significant relationships between *SPOP* expression and cytokine secretion (Enrichment score = 0.531, *P* < 0.001), T-cell activation (Enrichment score = 0.383, *P* = 0.040), T-cell differentiation (Enrichment score = 0.430, *P* = 0.032), and T-cell proliferation (Enrichment score = 0.455, *P* = 0.034). Furthermore, knockdown of SPOP resulted in elevated PD-L1 protein expression (Fig. S8B, *P* < 0.050), but had no effect on PD-L1 mRNA expression (Fig. S8A, *P* > 0.050).

*SPOP* mutations have been widely studied [36], and previous studies have reported somatic *SPOP* mutations in CRC [37, 38]. Apart from PD-L1, it is reported that the substrates of SPOP also include c-MYC [39], BRD2 [40], myeloid differentiation primary response gene 88 (MYD88) [41], interleukin enhancer binding factor 3 (ILF3) [42], GLI family zinc finger 2 (GLI2) [43], tumor protein p53 binding protein 1 (TP53BP1) [44], and SET domain containing 2 (SETD2) [45]. We obtained *SPOP* mutation and expression data from the Pan-Cancer Atlas project for CRC patients (Fig. S8C, *n* = 592). *CD274* mRNA expression was significantly increased in *SPOP* mutant CRC tissues (*P* < 0.05). However, the *SPOP* mutation did not lead to apparent changes in the expression levels of other substrates (*P* > 0.050). After YTHDF2 knockdown, protein expression of c-MYC and BRD2 did not show significant changes (Fig. S8D, *P* > 0.050). Besides, PD-L1 exhibited no significant changes upon SHOC2 knockdown (Fig. S8E, F, *P* > 0.050).

### YTHDF2 modulated *SPOP* mRNA in a m^6^A-dependent manner

**Fig. 6:**
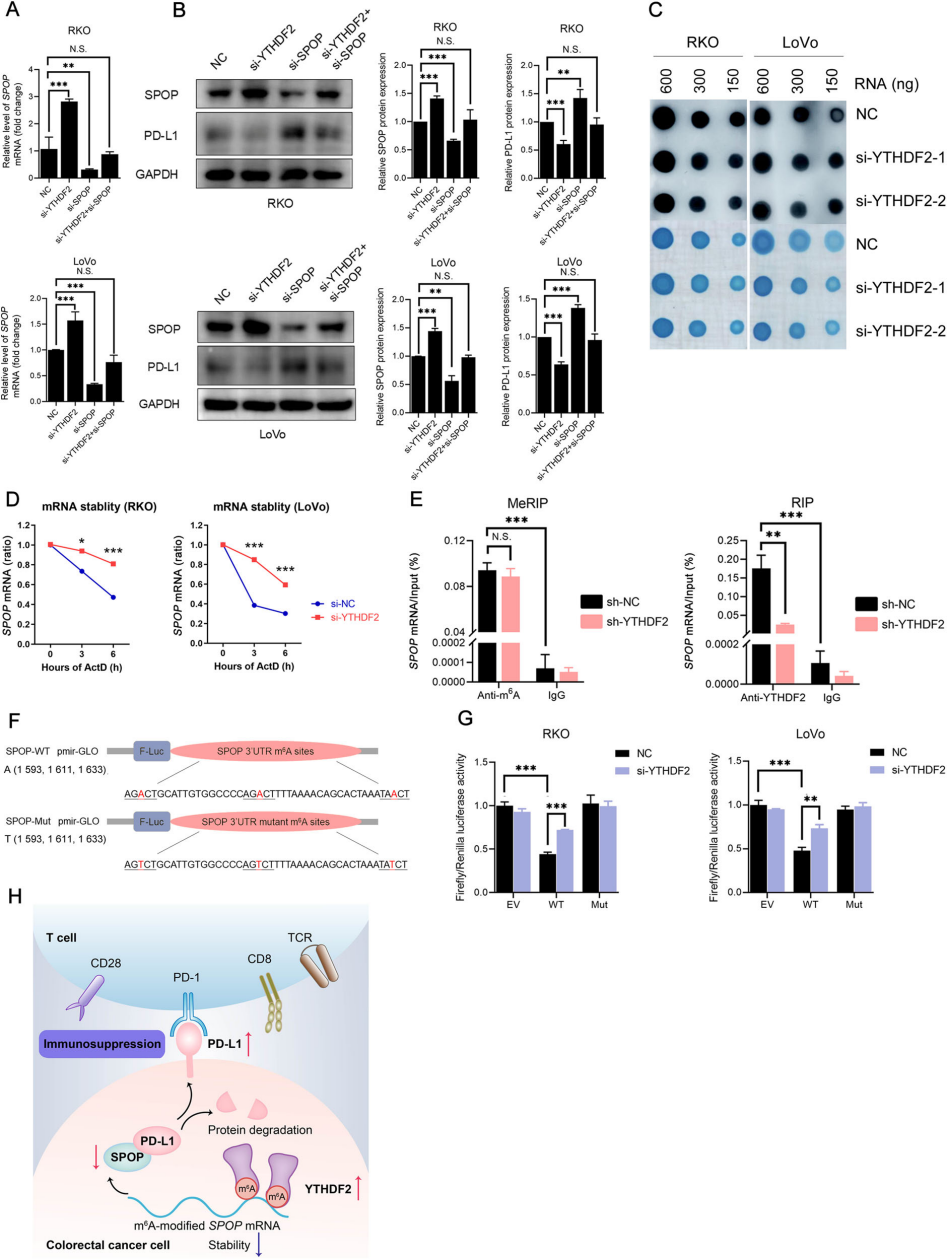
YTHDF2 modulated *SPOP* mRNA in a m^6^A-dependent manner. **A** qRT-PCR analysis of *SPOP* expression level in siRNAs (NC, si-YTHDF2, or si-SPOP) transfected CRC cell lines (RKO, upper; LoVo, lower). **B** Western blots (left) analysis of SPOP and PD-L1 expression level in siRNAs (NC, si-YTHDF2, or si-SPOP) transfected CRC cell lines (RKO, upper; LoVo, lower). The quantifications of SPOP (middle) and PD-L1 (right) were shown. **C** RNA m^6^A dot blot assay of siRNAs (NC or si-YTHDF2) transfected CRC cell lines (RKO and LoVo). Methylene blue staining served as the loading control. **D** The decay rate of *SPOP* mRNA was detected after 2 μmol/L ActD treatment at the indicated times through qRT-PCR. **E** MeRIP-qPCR (left) and RIP-qPCR (right) analysis of m^6^A levels and YTHDF2-binding ability in sh-NC or sh-YTHDF2 RKO cells. **F** Schematic showed the generation strategy for the pmirGLO luciferase reporters containing WT (upper) and Mut (lower; A to T) *SPOP* 3’UTR. **G** Luciferase activities of the indicated pmirGLO vector were measured in CRC cell lines (RKO, left; LoVo, right) with or without YTHDF2 knockdown. **H** A schematic diagram depicting the function of the YTHDF2-SPOP-PD-L1 axis in CRC. The hypothesis test for significance between two groups utilized the Student’s *t*-test. Results were analyzed with ANOVA for three or more groups. GAPDH was included as the loading control in western blots. HPRT1 served as the internal control in qRT-PCR. N.S., no statistical significance, *P < 0.050, **P < 0.010, and ***P < 0.001. Values are mean ± SEM. 3’UTR 3’ untranslated region, ActD actinomycin D, EV empty vector, F-Luc firefly/renilla luciferase, IgG immune globulin G, IP immunoprecipitation, m^6^A: N^6^-methyladenosine, MeRIP methylated RNA immunoprecipitation, Mut mutated-type; NC: negative control, RIP RNA binding protein immunoprecipitation, WT wild-type.

Based on the above evidence, YTHDF2 and SPOP had different effects on PD-L1 expression. The combination of siRNAs resulted in unchanged PD-L1 expression, indicating that SPOP knockdown could reverse the decline in PD-L1 induced by si-YTHDF2 (Fig. 6A, B, *P* > 0.050). Given that *SPOP* mRNA was identified as a target mRNA of YTHDF2, the next question was whether this impact relied on m^6^A methylation. A dot blot assay did not show an overall reduction in m^6^A modification after YTHDF2 knockdown in CRC cells (Fig. 6C). However, knockdown of YTHDF2 led to a significant increase in the lifespan of *SPOP* mRNA in RKO and LoVo cells (Fig. 6D, *P* < 0.050). Hence, YTHDF2 did not alter overall methylation levels but did affect the lifespan of its target mRNAs.

To further confirm the observed results, we performed MeRIP-qPCR and RIP-qPCR. Compared to the IgG group, the anti-m^6^A antibody cross-reacted with m^6^A-containing *SPOP* mRNA fragments, and the anti-YTHDF2 antibody successfully isolated the YTHDF2-mRNA complex (Fig. 6E, *P* < 0.001). As detected by m^6^A site-specific qPCR primers, *SPOP* mRNA levels were noticeably decreased after YTHDF2 knockdown in RIP-qPCR (*P* < 0.010), whereas there was no decrease observed in MeRIP-qPCR (*P* > 0.050). This result confirmed YTHDF2’s role as a reader in destabilizing mRNAs and directing transcripts to the decay machinery. The decrease in *SPOP* mRNA expression could be attributed to the binding of YTHDF2 to m^6^A sites located in the 3’UTR.

To prove that YTHDF2 functioned by binding directly to target mRNAs, we introduced mutations into the reporter transcript (Fig. 6F) and assessed the impact on mRNA abundance. The results showed a significant decrease in luciferase activity when m^6^A sites were introduced into the luciferase mRNA (Fig. 6G, *P* < 0.001). The overall luciferase activity increased following YTHDF2 knockdown, indicating a significant role of YTHDF2 in promoting mRNA degradation (*P* < 0.010). Conversely, mutations at these sites compromised YTHDF2’s ability to bind m^6^A sites. These results confirmed that YTHDF2 hindered *SPOP* expression in a m^6^A-dependent manner.

## Discussion

Previous studies have reported contradictory expression patterns of YTHDF2 during cancer progression. YTHDF2 is either upregulated [46] in acute myeloid leukemia or downregulated in melanoma [16] and CRC [47]. These results highlight the functional complexity of YTHDF2. In this study, we demonstrated that YTHDF2 was upregulated in CRC tissues, correlated with a poor prognosis, and closely associated with immune-related pathways (Fig. 1). YTHDF2 promoted CRC growth in vivo, and a higher count of CD4^+^ and CD8^+^ T cells was observed in the sh-YTHDF2 tumor (Fig. 2). High-throughput sequencing data and clinical sample data further supported a positive correlation between YTHDF2 and PD-L1. After YTHDF2 knockdown, the expression level and subsequent binding ability of PD-L1 were disrupted, potentially contributing to the relief of T-cell repression in the tumor microenvironment (Fig. 3). Mechanistically, YTHDF2 recognized m^6^A modifications within the 3’UTR of *SPOP* mRNA and promoted its degradation (Figs. 4–6). The consequent reduction in SPOP protein levels thereby facilitated the PD-L1/PD-1 immune checkpoint response. Thus, we developed the concept and described the function of the YTHDF2-SPOP-PD-L1 axis in CRC (Fig. 6H) to provide a basis for medical practice.

While PD-L1 is regulated at the transcription, post-transcription, translation, and post-translation levels [48], including the IFNγ signaling pathway [49], increasing evidence suggests that m^6^A modification plays a vital role in regulating PD-L1. The m^6^A writer METTL3, eraser FTO, and reader YTHDF1 have been shown to bind directly to PD-L1 mRNA in breast cancer [50], CRC [51], and prostate cancer [52]. Besides, IGF2BP3 can affect PD-L1 expression in the tumor microenvironment through lactic acid production [53]. Previous research has shown that YTHDF1 binds to mRNAs before YTHDF2 [54]. We demonstrated that YTHDF2, but not YTHDF1, affected PD-L1 expression in CRC (Fig. S5G, H). YTHDF2 primarily induces the decay of its target mRNAs, predicting a negative correlation with downstream gene expression [15]. Our observation of a positive correlation between YTHDF2 and PD-L1 protein levels, therefore, pointed to an indirect regulatory mechanism, distinct from the direct mRNA stabilization role reported for YTHDF1 in PD-L1 regulation [52]. RIP-seq data showed that the binding of YTHDF2 and *CD274* mRNA had no difference between the input and RIP groups (Fig. S7E), which indicated that YTHDF2 did not bind *CD274* mRNA directly. We found that YTHDF2 recognized m^6^A-dependent *SPOP* mRNA and mediated its degradation (Fig. 6D–G). As an adaptor protein for Cullin 3-based E3 ubiquitin ligases, SPOP has been shown to regulate of PD-L1 abundance through ubiquitin-mediated degradation in prostate cancer [34] and esophageal adenocarcinoma [55], which was consistent with our findings.

Although the expression level of PD-L1 in CRC tissues failed to predict treatment effectiveness, studies have revealed that PD-L1 expression is closely related to tumor stage and prognosis [56, 57]. Higher PD-L1 expression was more commonly observed in MSI-H tumors than in MSS tumors [58]. We also found higher *YTHDF2* and *CD274* expression in dMMR CRC (Fig. S3B). In addition, preliminary results have suggested that CRC patients with higher CD8^+^ T-cell infiltration would be more likely to benefit from immunotherapy [59]. Therefore, more studies are needed to determine whether altering PD-L1 expression profoundly affects CRC progression and treatment response.

In addition, highly selective YTHDF2 inhibitors show promising therapeutic potential in tumor treatment. A similar study reported that the use of Toll-like receptor 9 agonist-conjugated siRNAs specifically targeting YTHDF2 in tumor-associated macrophages enhanced the efficacy of anti-PD-L1 therapy [17]. Consistently, we also explored the connection between YTHDF2 knockdown and anti-PD-L1 treatment. The results showed that reduced YTHDF2 helped improve the treatment effect (Fig. S4). In addition, we found connections among *YTHDF2* expression, CMS status, and *KRAS* and *BRAF* mutations, indicating *YTHDF2*’s involvement in different pathological and molecular characteristics (Fig. S3). Moreover, researchers have exploited distinct inhibitors of m^6^A regulators as a form of combination therapy [60, 61].

However, we could not deny the study’s limitations. Given that microRNAs [62–64] and HIF-2α [65] have been found to regulate YTHDF2 expression, the cause of aberrant YTHDF2 expression in CRC remains unknown and deserves further investigation. Additionally, the understanding of interactions between immune cells is just the tip of the iceberg. Although we tried to mimic a humanized immune environment using a humanized mouse model, the PBMCs used were mainly composed of CD3^+^ T cells. Regarding the complex and dynamic nature of the immune response, it is important to rule out the impact of other immune cells. Apart from that, we failed to explore the function of somatic mutations in SPOP in CRC due to the low mutation frequency (< 5%) [19, 37], and more samples are needed for further analysis.

## Conclusions

The integration of the aforementioned results led us to propose a mechanism involving m^6^A-dependent *SPOP* mRNA degradation boosted by YTHDF2. The SPOP-mediated proteasome-dependent degradation of PD-L1 is weakened, promoting PD-L1 expression. Collectively, our results underscore the importance of YTHDF2 and suggest that combining targeting m^6^A regulators with anti-PD-1/PD-L1 therapy may be effective in treating CRC.

## Supplementary information

Supplementary materials

Original Western Blots

Original m6A and RIP peaks

## Data Availability

All data relevant to the study are included in the article or supplemental materials. Data are available upon reasonable request.
