# Supplementary material for: Reduced YTHDF2 inhibits PD-L1 expression by stabilizing m6A-containing SPOP mRNA in colorectal cancer
Source: Cell Death Dis. 2026 Mar 24;17(1):351. doi: 10.1038/s41419-026-08615-2 (PMC13040070; doi:10.1038/s41419-026-08615-2)
Supplement: Supplementary file 1 — Supplementary materials [file 41419_2026_8615_MOESM1_ESM.docx]

**Supplementary Materials**

Xian Xu^1,2,†^, Hao Chen^1,†^, Rongjie Zhao^1,†^, Jiansheng Xie^1^, Hao Liu^1^, Binbin Xie^1^, Jun Lou^1^, Haidong Wang^1^, Xinkai Wu^1^, Weidong Han^3,*^, Hongming Pan^1,*^, Jiaying Shen^1,*^

^1^Department of Medical Oncology, Sir Run Run Shaw Hospital, Zhejiang University School of Medicine, Hangzhou, Zhejiang, P. R. China.

^2^Department of Pulmonary and Critical Care Medicine, Sir Run Run Shaw Hospital, Zhejiang University School of Medicine, Hangzhou, Zhejiang, P. R. China.

^3^Department of Colorectal Medical Oncology, Zhejiang Cancer Hospital, Hangzhou, Zhejiang, P. R. China.

^†^These authors contributed equally to this work.

^*^Correspondence authors:

Dr. Jiaying Shen; Department of Medical Oncology; Sir Run Run Shaw Hospital; Zhejiang University School of Medicine; No. 3 East Qingchun Road; Hangzhou; Zhejiang; P.R. China; 310016; Email: 11318167@zju.edu.cn.

Dr. Hongming Pan; Department of Medical Oncology; Sir Run Run Shaw Hospital; Zhejiang University School of Medicine; No. 3 East Qingchun Road; Hangzhou; Zhejiang; P.R. China; 310016; Email: panhongming@zju.edu.cn.

Dr. Weidong Han; Department of Colorectal Medical Oncology, Zhejiang Cancer Hospital; No. 1, East Banshan Road; Hangzhou; Zhejiang; P.R. China; 310022; Email: hanwd@zju.edu.cn.

Email addresses for all authors:

Xian Xu, xxian@zju.edu.cn.

Hao Chen, 22218189@zju.edu.cn.

Rongjie Zhao, rogerzhao@zju.edu.cn.

Jiansheng Xie, xiejiansheng@zju.edu.cn.

Hao Liu, liuhao431@163.com.

Binbin Xie, xiebb@zju.edu.cn.

Jun Lou, loujundz@163.com

Haidong Wang, haidongwang@zju.edu.cn.

Xinkai Wu, wuxinkai@zju.edu.cn

Weidong Han, hanwd@zju.edu.cn.

Hongming Pan, panhongming@zju.edu.cn.

Jiaying Shen, 11318167@zju.edu.cn.


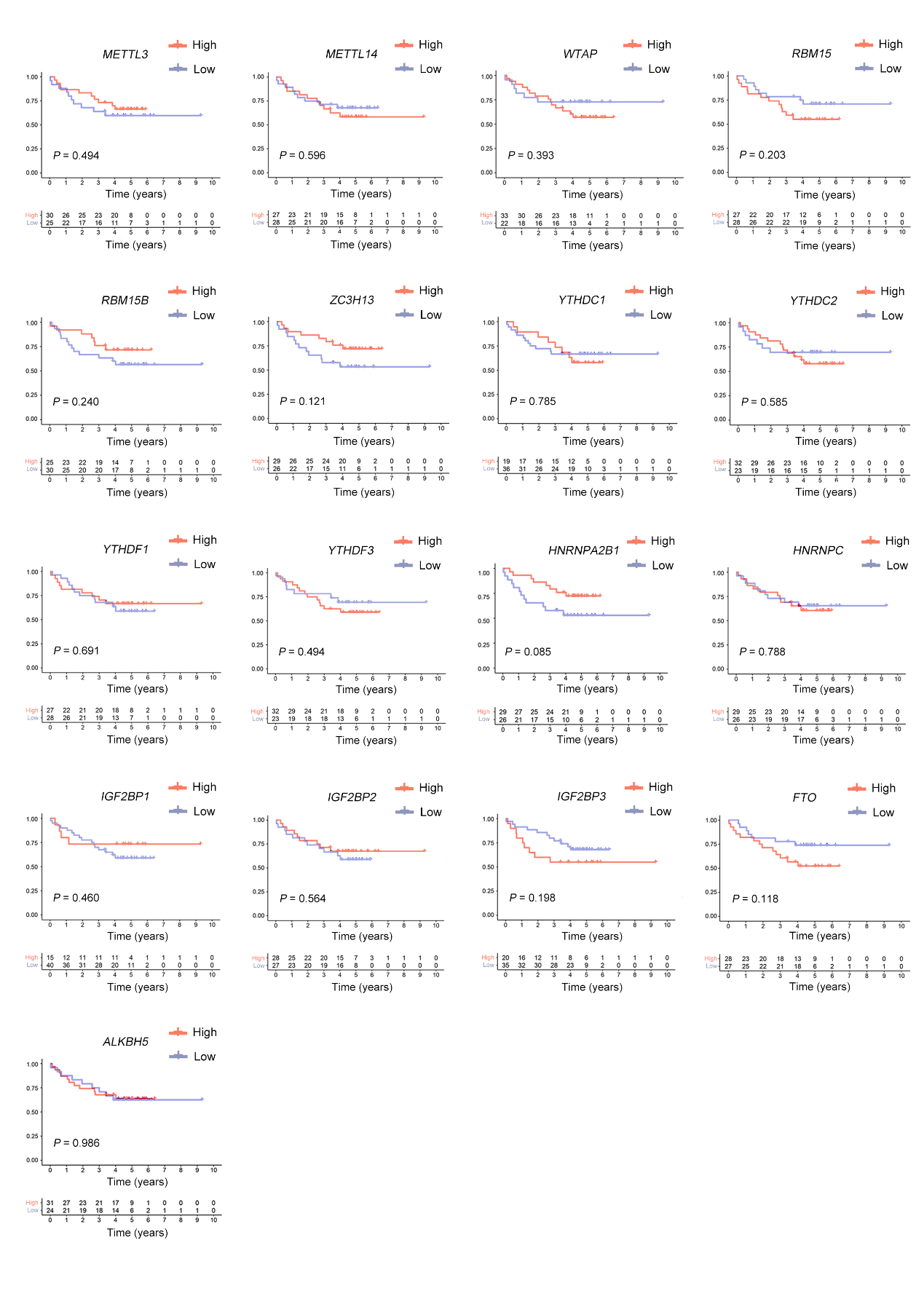


**Supplementary Figure S1. Part of m^6^A regulators did not play important roles in affecting the prognosis of CRC, related to Figure 1.**

Kaplan-Meier analysis of OS analysis for patients with high or low m^6^A RNA regulators expression levels from GSE17537 (*n* = 55). The top half of figure showed the survival curves and the bottom half of figure represented the number of patients at different points of time. Red represented high expression level whereas blue represented low expression level.


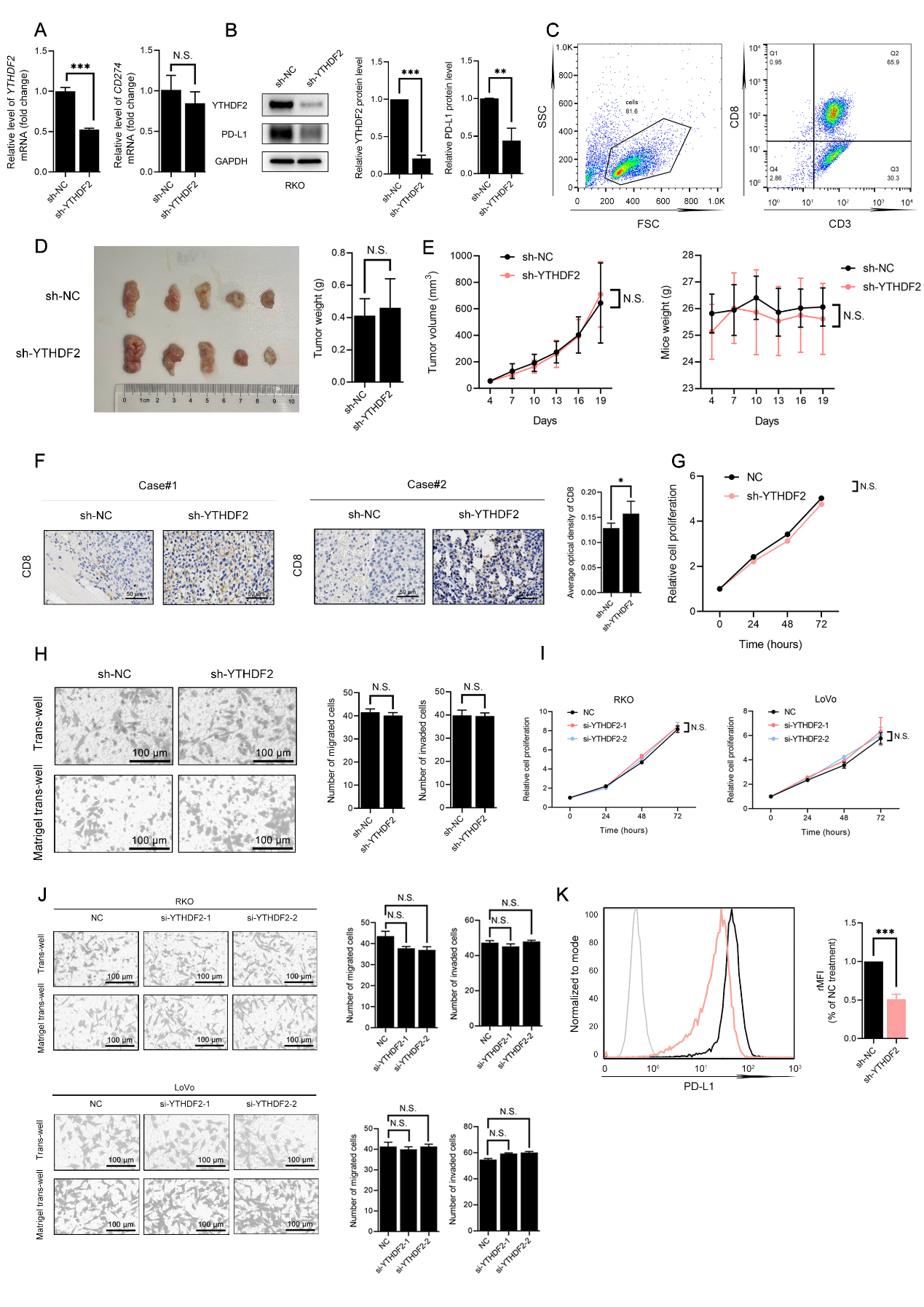


**Supplementary Figure S2. YTHDF2 was not related to cell proliferation, migration, or invasion, related to Figure 2.**

A. qRT-PCR analysis of *YTHDF2* and *CD274* expression levels post lentivirus-mediated transfection of sh-NC or sh-YTHDF2 in RKO cells. HPRT1 served as the internal control.

B. Western blots (left) analysis of YTHDF2 and PD-L1 expression levels post lentivirus-mediated transfection of sh-NC or sh-YTHDF2 in RKO cells. GAPDH was included as the loading control. The quantifications of YTHDF2 (middle) and PD-L1 (right) were shown.

C. Flow cytometric analysis of PBMCs before injected to the caudal vein of NSG mice.

D-E. Representative images of subcutaneous tumors formed by sh-NC or sh-YTHDF2 RKO cells without PBMCs injection (D, left). Tumor masses were weighed after harvesting (D, right). The tumor volumes (E, left) and mice weights (E, right) were monitored after cell implanting every 3 days (*n* = 5).

F. Representative image (left, middle) and quantification (right) of CD8 staining in subcutaneous tumors. Scale bars, 50 μm.

G. CCK-8 proliferation assay in sh-NC or sh-YTHDF2 RKO cells.

H. Representative images of trans-well migration and invasion assays in sh-NC or sh-YTHDF2 RKO cells (left). The quantification was shown (right).

I. CCK-8 proliferation assay in siRNAs (NC or si-YTHDF2) transfected CRC cell lines (RKO, left; LoVo, right).

J. Representative images of trans-well migration and invasion assays in RKO (upper) and LoVo cells (lower) after YTHDF2 knockdown (left). The quantification was shown (right).

K. PD-L1 was visualized by flow cytometry (left) in sh-NC or sh-YTHDF2 RKO cells. The quantification was shown (right).

The hypothesis test for significance between two groups utilized the Student’s t-test. Results were analyzed with ANOVA for three or more groups. N.S., no statistical significance, **P* < 0.050, ***P* < 0.010, and ****P* < 0.001. Values are mean ± SEM.

Abbreviations: MFI: mean fluorescence intensity; NC: negative control.

**
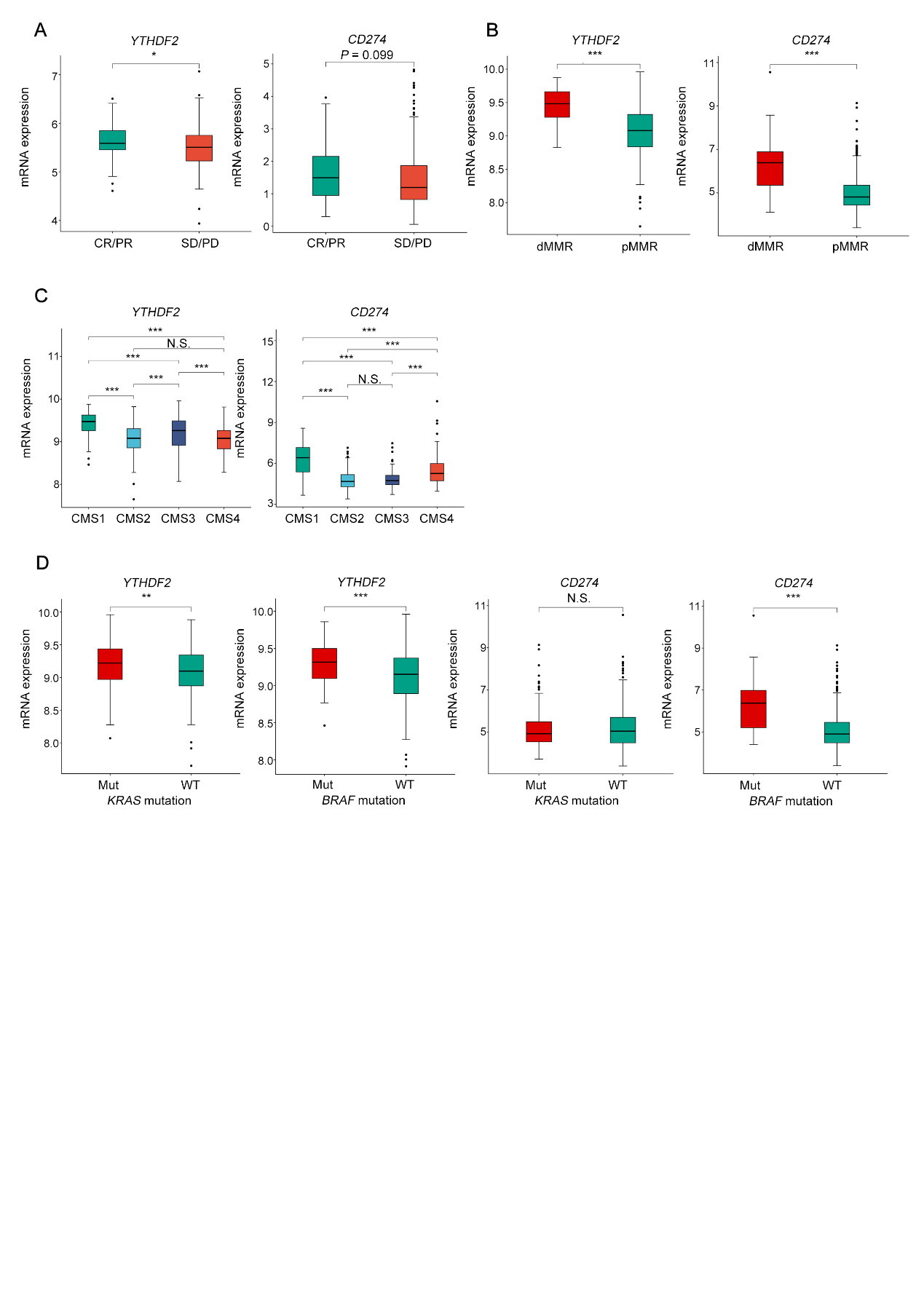
**

**Supplementary Figure S3. Expression patterns of *YTHDF2* in CRC with different pathological and molecular characteristics, related to Figure 2.**

A. The transcription profile according to PD-L1 blockade atezolizumab treatment response in the IMvigor210 cohort (*n* = 348). CR and PR were defined as treatment-sensitive, while SD and PD were treatment-resistant.

B-C. The expression of *YTHDF2* and *CD274* in (B) dMMR/pMMR (75 dMMR and 444 pMMR) and (C) CMS (76 CMS1, 182 CMS2, 90 CMS3, and 128 CMS4) classification in GSE39582.

D. The relationship between *YTHDF2*, *CD274* expression and conditions of *KRAS* mutation (217 *KRAS*-mutant and 328 *KRAS*-wild type), *BRAF* mutation (51 *BRAF*-mutant and 461 *BRAF*-wild type).

Wilcox test was adopted. N.S., no statistical significance, **P* < 0.050, ** *P* < 0.010, and *** *P* < 0.001.

Abbreviations: CMS: consensus molecular subtypes; CR: complete response; dMMR: deficient mismatch repair; Mut: mutated-type; PD: progressive disease; pMMR: mismatch repair-proficient; PR: partial response; SD: stable disease; WT: wild-type.

**
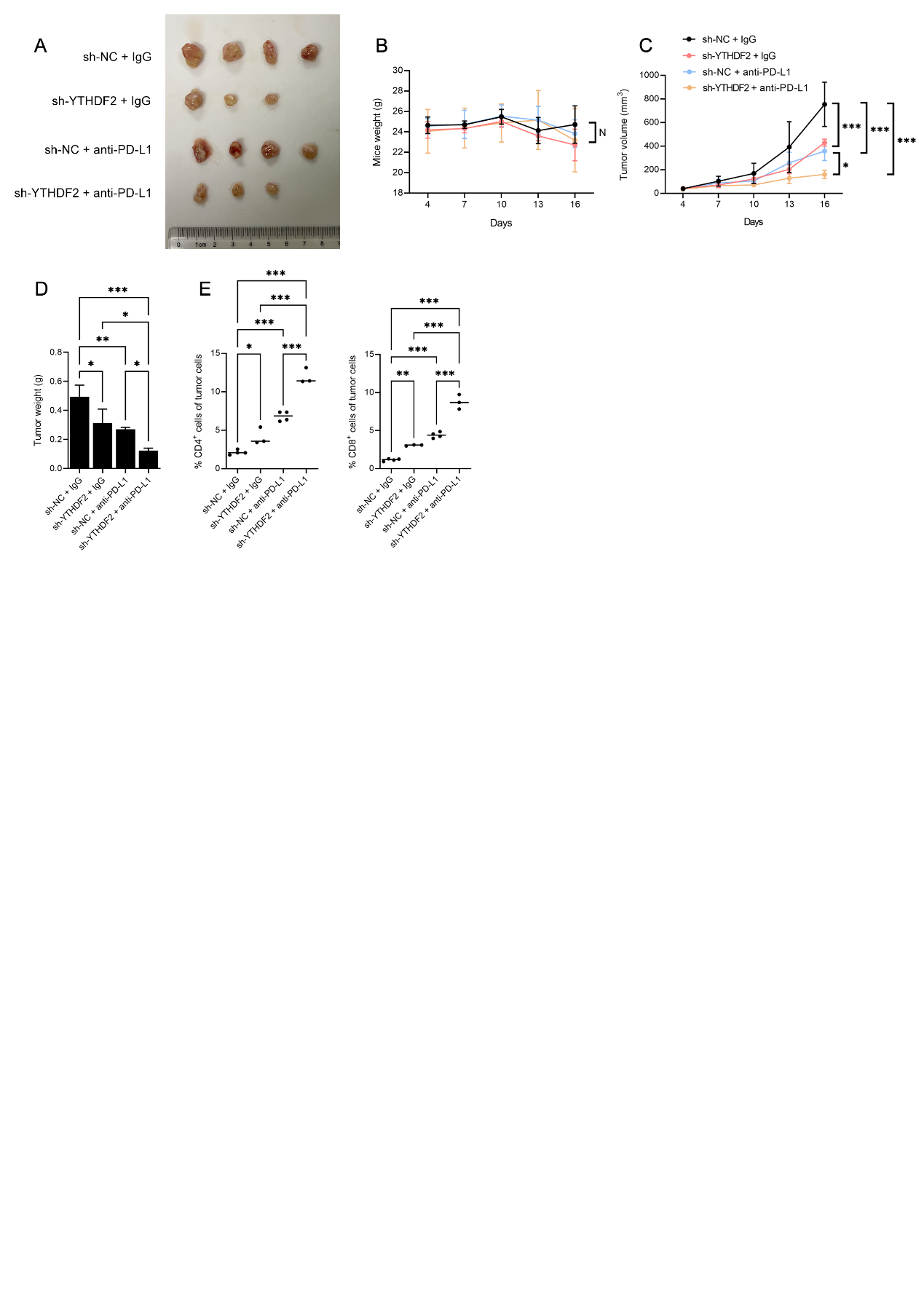
**

**Supplementary Figure S4. Anti-PD-L1 treatment with YTHDF2 knockdown inhibited tumor growth, related to Figure 2.**

A. Representative images of subcutaneous tumors formed by sh-NC or sh-YTHDF2 RKO cells treated with IgG or anti-PD-L1 antibody (*n* = 3~4).

B-C. The mice weights (B) and tumor volumes (C) were monitored after cell implanting every 3 days (*n* = 3~4).

D. Tumor masses were weighed after harvesting.

E. Flow cytometric analysis of the percentage of CD4^+^ or CD8^+^ tumor infiltrating-lymphocytes.

The hypothesis test for significance between two groups utilized the Student’s t-test. Results were analyzed with ANOVA for three or more groups. N.S., no statistical significance, **P* < 0.050, ***P* < 0.010, and ****P* < 0.001. Values are mean ± SEM.

Abbreviations: IgG: immune globulin G; NC: negative control.

**
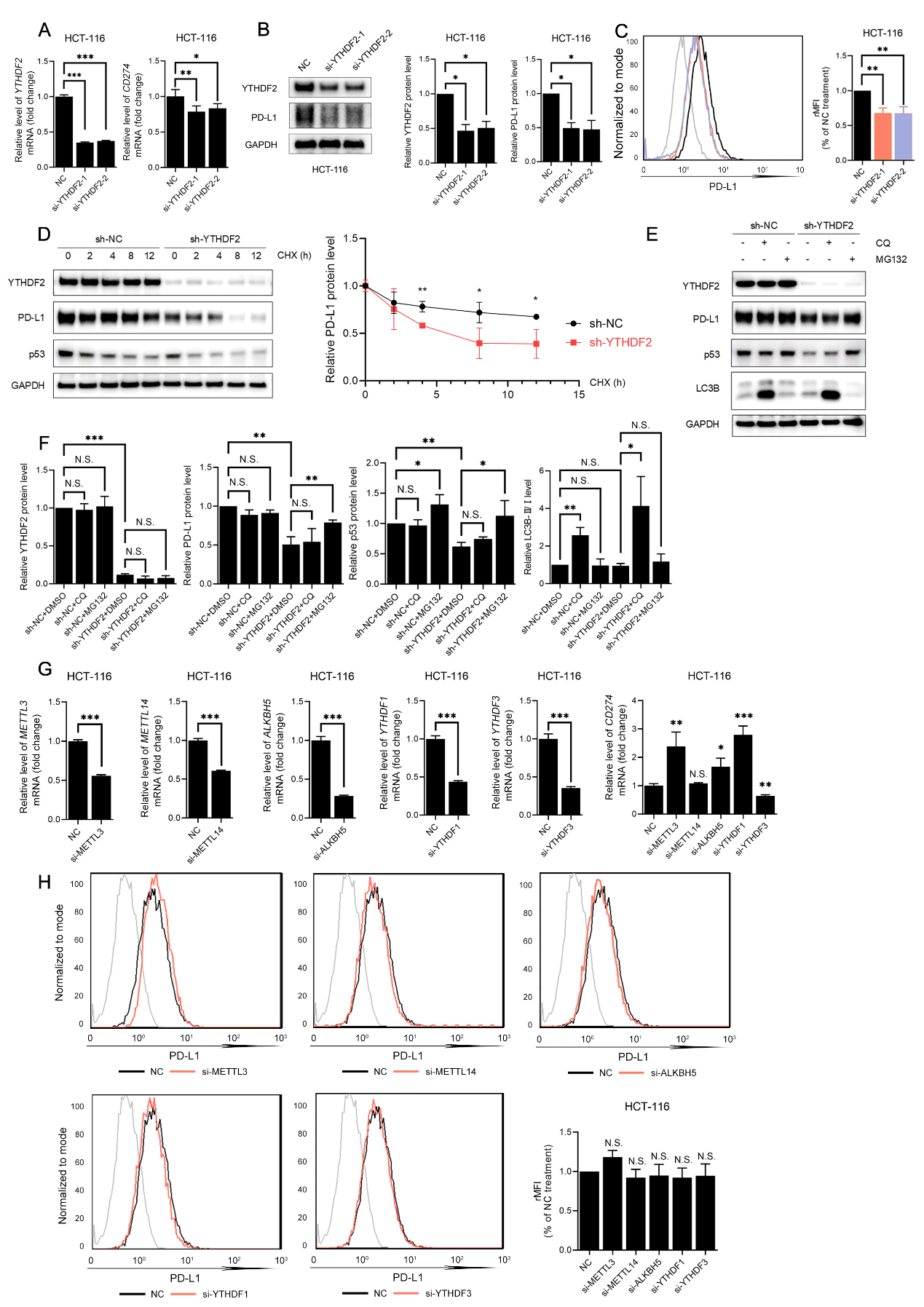
**

**Supplementary Figure S5. Reduced YTHDF2 decreased PD-L1 expression, related to Figure 3.**

A. qRT-PCR analysis of *YTHDF2* and *CD274* expression levels post siRNAs (NC or si-YTHDF2) transfection in CRC cell line (HCT-116).

B. Western blots (left) analysis of YTHDF2 and PD-L1 expression levels post siRNAs (NC or si-YTHDF2) transfection in CRC cell line (HCT-116). The quantifications of YTHDF2 (middle) and PD-L1 (right) were shown.

C. PD-L1 was visualized by flow cytometry (left) in HCT-116 cells treated with siRNAs (NC or si-YTHDF2). The quantification was shown (right).

D. CHX chase assay (10 μM) showing the degradation of PD-L1 in sh-NC and sh-YTHDF2 RKO cells. Western blots (left) and the quantification (right) showing PD-L1 protein level.

E-F. Western blots (E) showing PD-L1 protein levels in sh-NC and sh-YTHDF2 RKO cells after treatment with CQ (25 μM) or MG132 (5 μM). The quantification was shown (F).

G. qRT-PCR analysis of m^6^A RNA regulators and *CD274* expression levels post siRNAs transfection in CRC cell line (HCT-116).

H. PD-L1 was visualized by flow cytometry in HCT-116 cells treated with m^6^A RNA regulators siRNAs. The quantification was shown (lower, right).

The hypothesis test for significance between two groups utilized the Student’s t-test. Results were analyzed with ANOVA for three or more groups. GAPDH was included as the loading control in western blots. HPRT1 served as the internal control in qRT-PCR. N.S., no statistical significance, **P* < 0.050, ***P* < 0.010, and ****P* < 0.001. Values are mean ± SEM.

Abbreviations: CHX: cycloheximide; CQ: chloroquine; MFI: mean fluorescence intensity; NC: negative control.

**
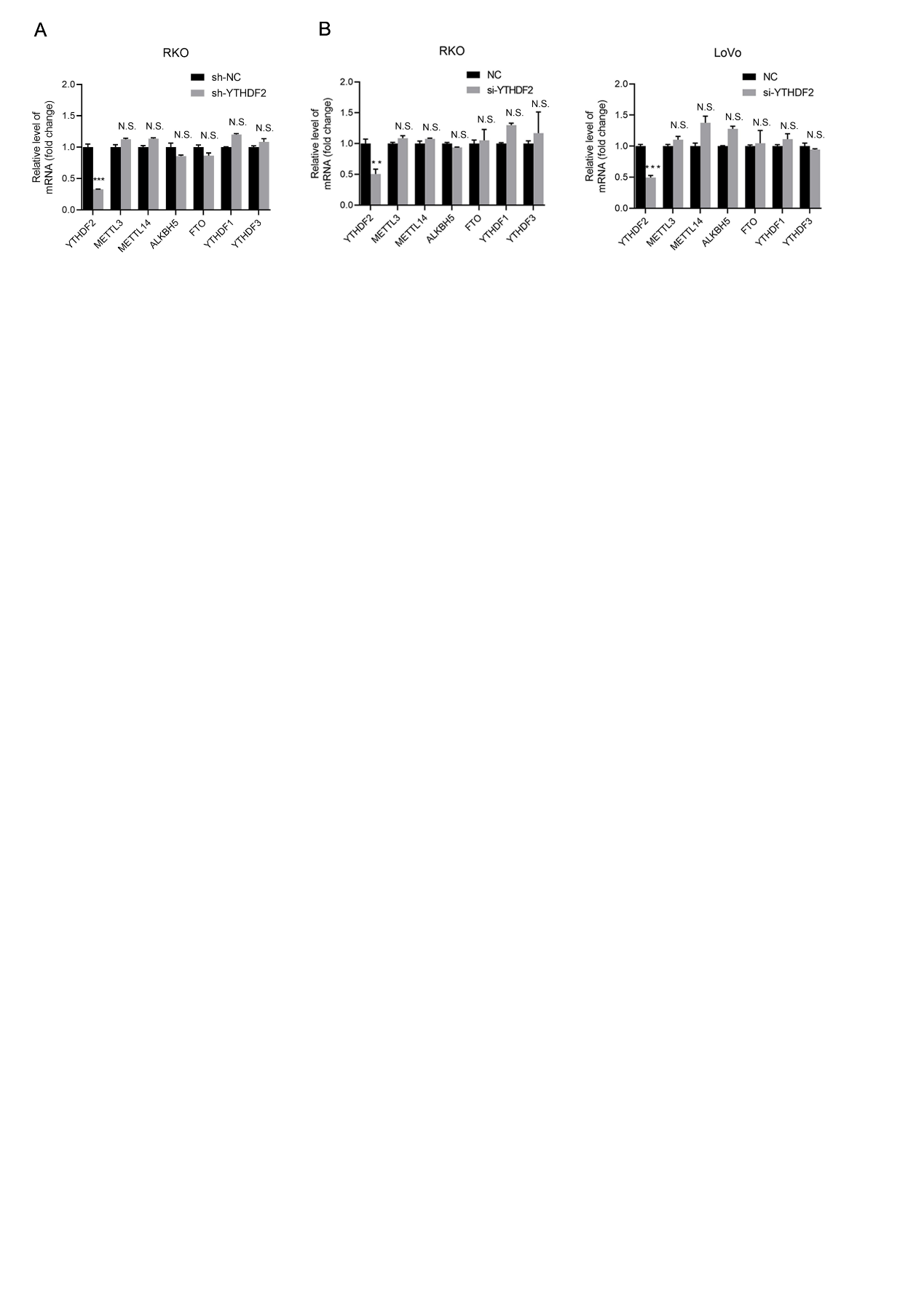
**

**Supplementary Figure S6. Knockdown of YTHDF2 did not impact the expression levels of other m^6^A regulators, related to Figure 3.**

A. qRT-PCR analysis m^6^A RNA regulators expression in sh-NC or sh-YTHDF2 RKO cells.

B. qRT-PCR analysis m^6^A RNA regulators expression in siRNAs (NC or si-YTHDF2) transfected CRC cell lines (RKO, left; LoVo, right).

The hypothesis test for significance between two groups utilized the Student’s t-test. HPRT1 served as the internal control in qRT-PCR. N.S., no statistical significance, **P* < 0.050, ***P* < 0.010, and ****P* < 0.001. Values are mean ± SEM.

Abbreviations: NC: negative control.

**
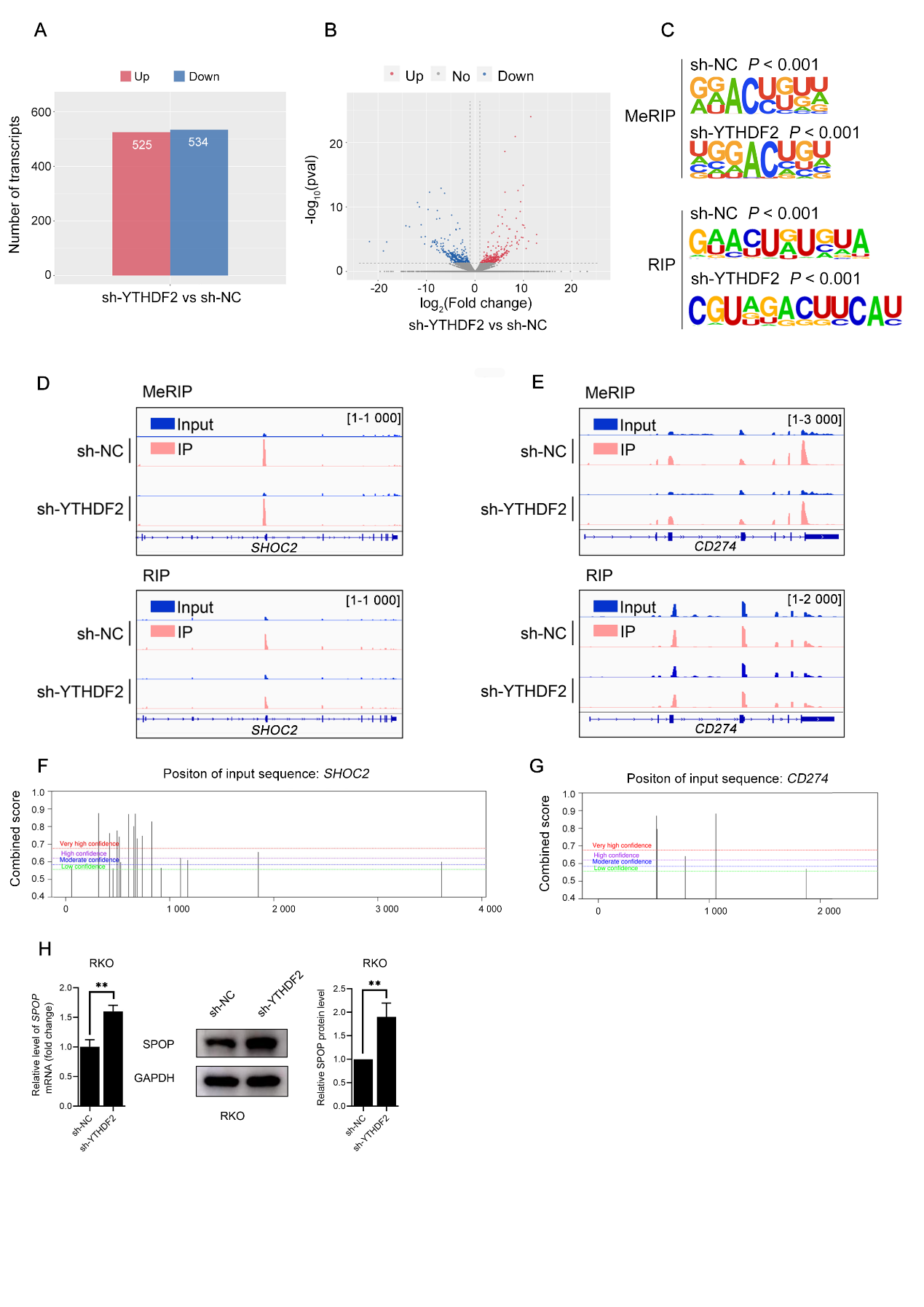
**

**Supplementary Figure S7. Identification of potential target mRNAs of YTHDF2, related to Figure 4.**

A. Differentially expressed transcripts were shown by analysis of RNA-seq data in sh-NC and sh-YTHDF2 RKO cells. Red represented up-regulated transcripts whereas blue represented down-regulated transcripts.

B. Volcano plot of differentially expressed transcripts. Significantly up-regulated or down-regulated transcripts were plotted in red and blue points respectively.

C. Motif analysis by the HOMER motif discovery tool revealed a “DRACH” consensus (where D represents A, G, or U, R represents A or G, H represents A, C, or U) for m^6^A modification sites (upper) and YTHDF2-binding sites (lower).

D-E. IGV tracks displayed the distribution of m^6^A peaks (upper) and YTHDF2-binding (lower) sites along *SHOC2* (D) and *CD274* (E) mRNAs according to MeRIP-seq and RIP-seq data. The abscissa represented transcripts, and the ordinate represented peaks signals. Red represented IP group whereas blue represented Input group.

F-G. The potential m^6^A sites of *SHOC2* (F) and *CD274* (G) mRNA were predicted by SRAMP. The abscissa represented transcripts, and the ordinate represented combined scores Different color lines indicated different confidences (red, purple, blue and green respectively represented very high, high, moderate and low confidence).

H. qRT-PCR (left) and western blots (middle) analysis of SPOP expression levels in sh-NC or sh-YTHDF2 RKO cells. The quantification was shown (right).

The hypothesis test for significance between two groups utilized the Student’s t-test. GAPDH was included as the loading control in western blots. HPRT1 served as the internal control in qRT-PCR. N.S., no statistical significance, **P* < 0.050, ** *P* < 0.010, and *** *P* < 0.001. Values are mean ± SEM.

Abbreviations: IP: immunoprecipitation; MeRIP: methylated RNA immunoprecipitation; NC: negative control; RIP: RNA binding protein immunoprecipitation.

**
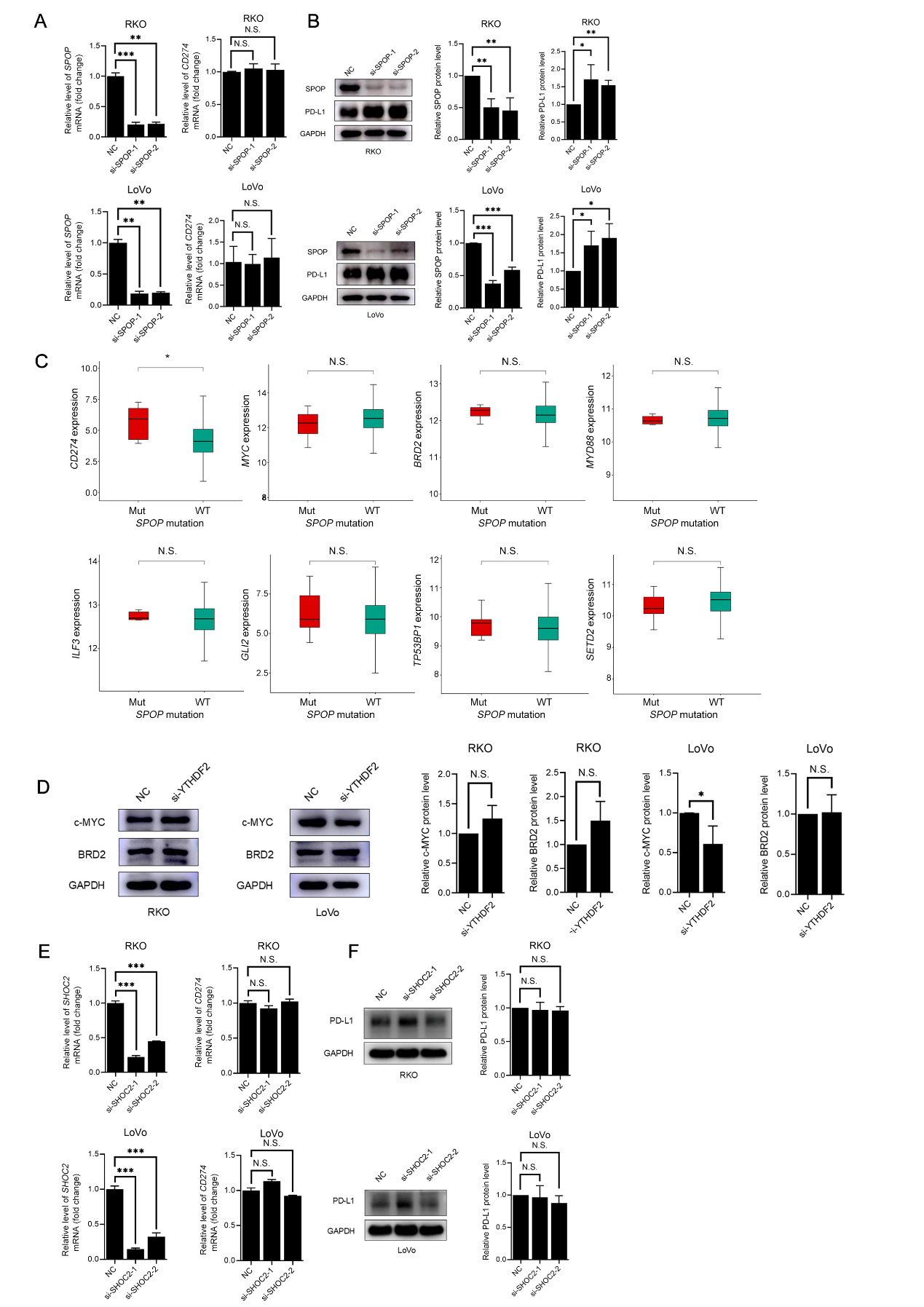
**

**Supplementary Figure S8. YTHDF2-mediated *SPOP* mRNA decay did not affect the stability of other substrates in CRC, related to Figure 5.**

A. qRT-PCR analysis of *SPOP* and *CD274* in siRNAs (NC or si-SPOP) transfected CRC cell lines (RKO, upper; LoVo, lower).

B. Western blots (left) analysis of SPOP and PD-L1 in siRNAs (NC or si-SPOP) transfected CRC cell lines (RKO, upper; LoVo, lower). The quantifications of SPOP (middle) and PD-L1 (right) were shown.

C. *CD274* expression levels were compared in 7 *SPOP*-mutant and 585 *SPOP*-wild type CRC tissues. Wilcox test was adopted.

D. Western blots (left) analysis of c-MYC and BRD2 in siRNAs (NC or si-YTHDF2) transfected CRC cell lines (RKO and LoVo). The quantification of western blots was shown (right).

E. qRT-PCR analysis of *SHOC2* and *CD274* in siRNAs (NC or si-SHOC2) transfected CRC cell lines (RKO, upper; LoVo, lower).

F. Western blots (left) analysis of PD-L1 in siRNAs (NC or si-SHOC2) transfected CRC cell lines (RKO, upper; LoVo, lower). The quantification of western blots was shown (right).

The hypothesis test for significance between two groups utilized the Student’s t-test. Results were analyzed with ANOVA for three or more groups. GAPDH was included as the loading control in western blots. HPRT1 served as the internal control in qRT-PCR. N.S., no statistical significance, **P* < 0.050, ***P* < 0.010, and ****P* < 0.001. Values are mean ± SEM.

Abbreviations: Mut: mutated-type; NC: negative control; WT: wild-type.

**Table S1. siRNAs and sh-RNAs used for silencing genes.**

|  | **Sequence** | |
| --- | --- | --- |
|  | **Sense 5’→3’** | **Antisense 5’→3’** |
| si-NC | UUCUCCGAACGUGUCACGUTT | ACGUGACACGUUCGGAGAATT |
| si-YTHDF2-1 | GCCCAAUAAUGCAUAUACUTT | AGUAUAUGCAUUAUUGGGCTT |
| si-YTHDF2-1 | GCUCUGGAUAUAGUAGCAATT | UUGCUACUAUAUCCAGAGCTT |
| si-METTL3 | GCAAGUAUGUUCACUAUGATT | UCAUAGUGAACAUACUUGCTT |
| si-METTL14 | GGCUAAAGGAUGAGUUAAUTT | AUUAACUCAUCCUUUAGCCTT |
| si-YTHDF1 | CCGCGUCUAGUUGUUCAUGAATT | UUCAUGAACAACUAGACGCGGTT |
| si-YTHDF3 | AUGGAUUAAAUCAGUAUCUAATT | UUAGAUACUGAUUUAAUCCAUTT |
| si-ALKBH5 | ACAAGUACUUCUUCGGCGATT | UCGCCGAAGAAGUACUUGUTT |
| si-SPOP-1 | CAACUAUCAUGCUUCGGAUTT | AUCCGAAGCAUGAUAGUUGTT |
| si-SPOP-2 | GGUAAAGGUUCCUGAGUGCTT | GCACUCAGGAACCUUUACCTT |
| si-SHOC2-1 | AAGCUGCGGAUGCUUGAUUTT | AAUCAAGCAUCCGCAGCUUTT |
| si-SHOC2-2 | UACCUUCGCUUUAAUCGUAUATT | UAUACGAUUAAAGCGAAGGUATT |
| sh-NC | TTCTCCGAACGTGTCACGT |  |
| sh-YTHDF2 | TTCCTACCAGATGCAATGTTT |  |

**Table S2. Primers for qPCR analysis.**

| **Gene** | **Sequence** | |
| --- | --- | --- |
|  | **Forward 5’→3’** | **Reverse 5’→3’** |
| METTL3 | AAGCTGCACTTCAGACGAAT | GGAATCACCTCCGACACTC |
| METTL14 | AGAAACTTGCAGGGCTTCCT | TCTTCTTCATATGGCAAATTTTCTT |
| YTHDF1 | ATGTCGGCCACCAGCGTGGACA | TCATTGTTTGTTTCGACTCTGC |
| YTHDF2 | GGCAGCACTGAAGTTGGG | CTATTGGAAGCCACGATGTTA |
| YTHDF3 | TGACAACAAACCGGTTACCA | TGTTTCTATTTCTCTCCCTACGC |
| ALKBH5 | CCCGAGGGCTTCGTCAACA | CGACACCCGAATAGGCTTGA |
| FTO | TGGGTTCATCCTACAACGG | CCTCTTCAGGGCCTTCAC |
| CD274 | CTGTCACGGTTCCCAAGGAC | GACAATTAGTGCAGCCAGGTC |
| HPRT1 | TGACACTGGCAAAACAATGCA | GGTCCTTTTCACCAGCAAGCT |
| SPOP | GCCGCTCTCAGAGATTTCCT | AGCACCAACTCTCAGCTACG |
| SHOC2 | GGGGCTCCTGACGGAACT | TGGATCTAAAAGCACTATGCCC |
| SPOP-m^6^A | TGTTGTGAGGGGAAGAGACTGC | CCCCCGTTTCCCCCAAGTTATT |

**Table S3. Wild-type and mutant-type m^6^A sequences in SPOP 3’UTR.**

| **SPOP 3’UTR** | **Sequence 5’→3’** |
| --- | --- |
| Wild-type  (1 571-1 660 bp) | ACTGTTGCTGCCACTGACCACCAGGTAGACAGCGCAATCTGTGGAGCTTTTACTCTGTT  GTGAGGGGAAGAGACTGCATTGTGGCCCCAGACTTTTAAAACAGCACTAAATAACTTG  GGGGAAACGGGGGGAGGGAAAATGAAATGAAAACCCTGTTGCTGCGTCACTGTGTTCC  CTTTGGCCTGGCTGAGTTTGATACTGTGGGGATTC |
| Mutant-type  (1 571-1 660 bp) | ACTGTTGCTGCCACTGACCACCAGGTAGACAGCGCAATCTGTGGAGCTTTTACTCTGTT  GTGAGGGGAAGAGTCTGCATTGTGGCCCCAGTCTTTTAAAACAGCACTAAATATCTTGG  GGGAAACGGGGGGAGGGAAAATGAAATGAAAACCCTGTTGCTGCGTCACTGTGTTCCC  TTTGGCCTGGCTGAGTTTGATACTGTGGGGATTC |

**Table S4. Primers for PCR and vector construction.**

| **Primer** | | **Sequence 5’→3’** |
| --- | --- | --- |
| SPOP 3’UTR | Forward | AACGAGCTCGCTAGCCTCGAGACTGTTGCTGCCACTGACCAC |
|  | Reverse | CAGGTCGACTCTAGACTCGAGGAATCCCCACAGTATCAAACTCAGC |
| SPOP mutant 3’UTR | Forward | GGCCCCAGTCTTTTAAAACAGCACTAAATATCTTGGGGGAAACGGGGGG |
|  | Reverse | TTTAAAAGACTGGGGCCACAATGCAGACTCTTCCCCTCACAACAGAGTAAAA |
