# Supplementary material for: Reduced YTHDF2 inhibits PD-L1 expression by stabilizing m6A-containing SPOP mRNA in colorectal cancer
Source: Cell Death Dis. 2026 Mar 24;17(1):351. doi: 10.1038/s41419-026-08615-2 (PMC13040070; doi:10.1038/s41419-026-08615-2)
Supplement: Supplementary file 2 — Original Western Blots [file 41419_2026_8615_MOESM2_ESM.docx]

Figure 1. Original western blots for Figure 2G.


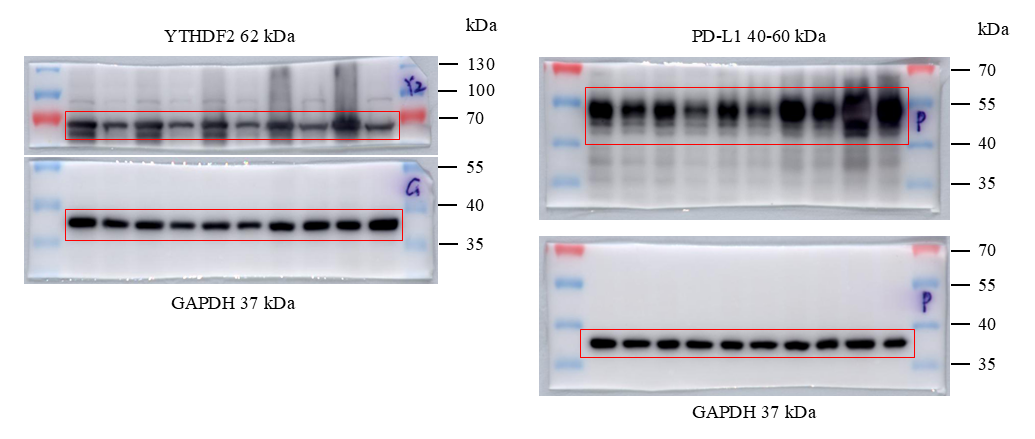


Figure 1 shows the whole blot after cutting membrane at molecular weight 62 kDa, 40-60 kDa and 37 kDa for YTHDF2 (62 kDa), PD-L1 (40-60 kDa) and GAPDH (37 kDa).

Figure 2. Original western blots for Figure 3B.


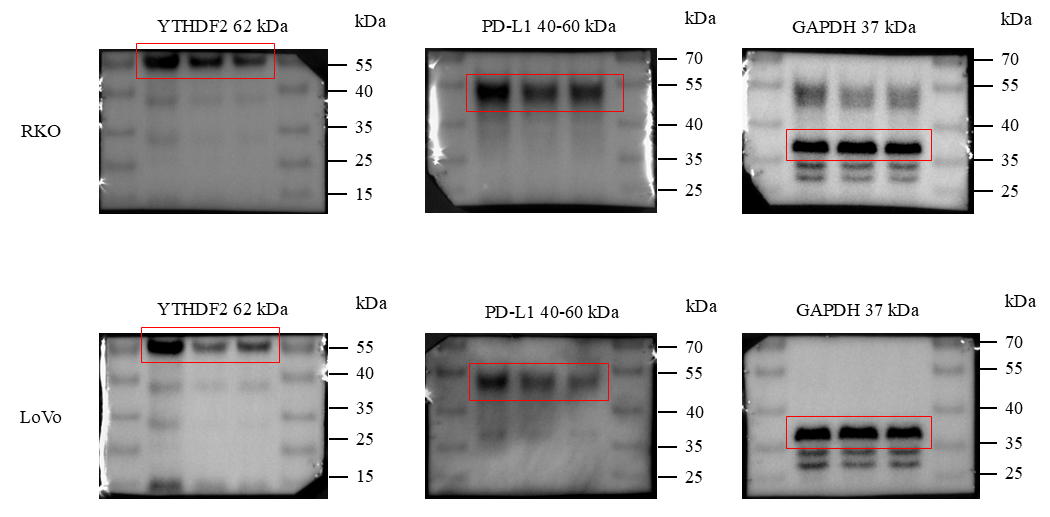


Figure 2 shows the whole blot after cutting membrane at molecular weight 62 kDa, 40-60 kDa and 37 kDa for YTHDF2 (62 kDa), PD-L1 (40-60 kDa) and GAPDH (37 kDa).

Figure 3. Original western blots for Figure 3E.


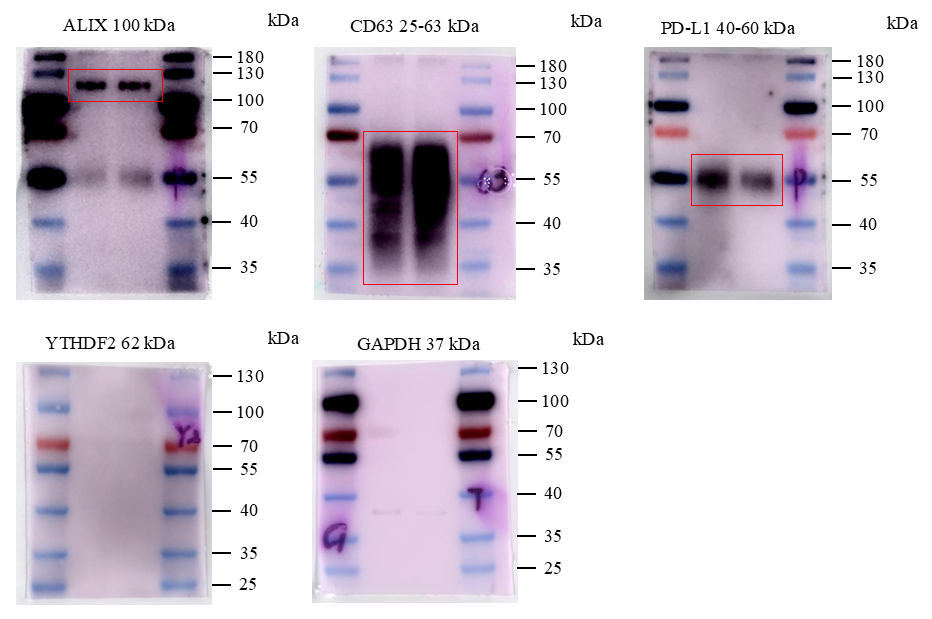


Figure 3 shows the whole blot after cutting membrane at molecular weight 100 kDa, 25-63 kDa, 40-60 kDa, 62 kDa and 37 kDa for ALIX (100 kDa), CD63 (25-63 kDa), PD-L1 (40-60 kDa), YTHDF2 (62 kDa) and GAPDH (37 kDa).

Figure 4. Original western blots for Figure 5A.


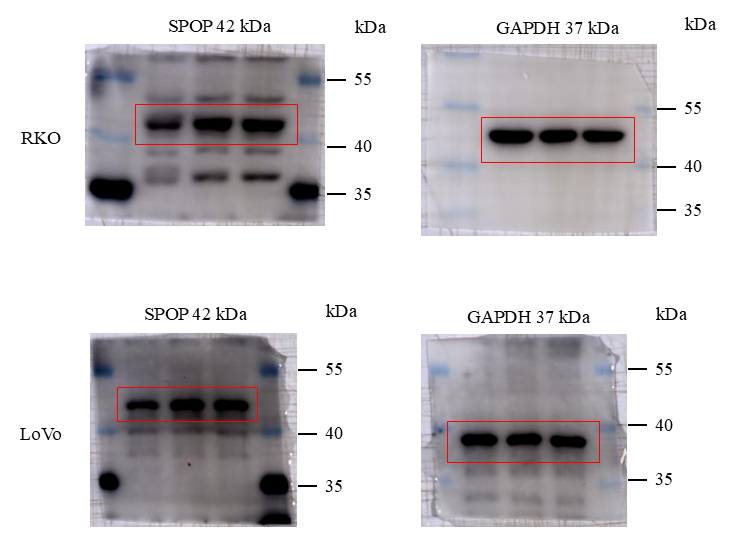


Figure 4 shows the whole blot after cutting membrane at molecular weight 42 kDa and 37 kDa for SPOP (42 kDa) and GAPDH (37 kDa).

Figure 5. Original western blots for Figure 6B.


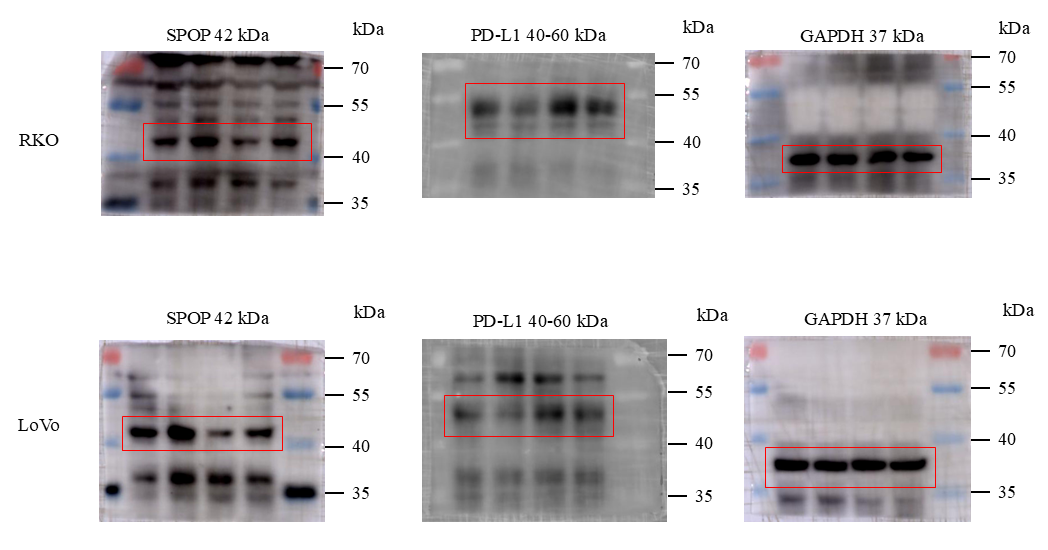


Figure 5 shows the whole blot after cutting membrane at molecular weight 42 kDa, 40-60 kDa and 37 kDa for SPOP (42 kDa), PD-L1 (40-60 kDa) and GAPDH (37 kDa).

Figure 6. Original western blots for Figure S2B.


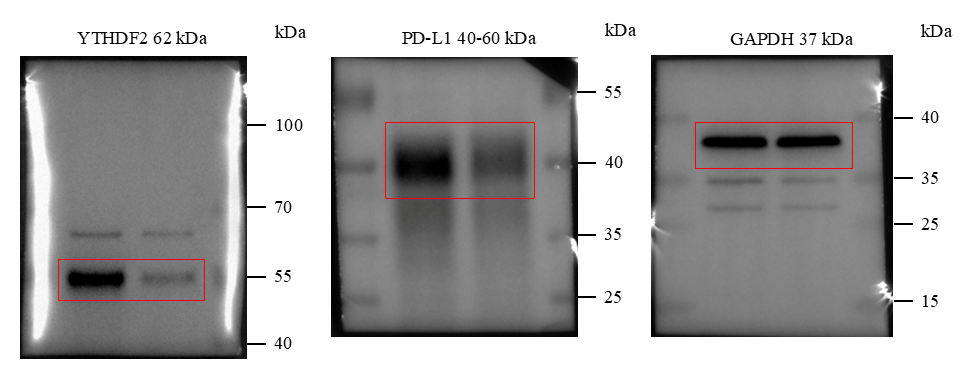


Figure 6 shows the whole blot after cutting membrane at molecular weight 62 kDa, 40-60 kDa and 37 kDa for YTHDF2 (62 kDa), PD-L1 (40-60 kDa) and GAPDH (37 kDa).

Figure 7. Original western blots for Figure S5B.


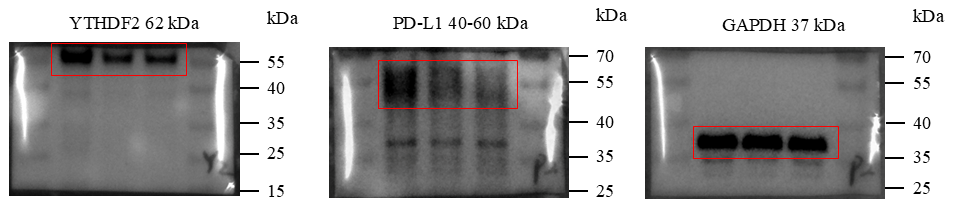


Figure 7 shows the whole blot after cutting membrane at molecular weight 62 kDa, 40-60 kDa and 37 kDa for YTHDF2 (62 kDa), PD-L1 (40-60 kDa) and GAPDH (37 kDa).

Figure 8. Original western blots for Figure S5D.


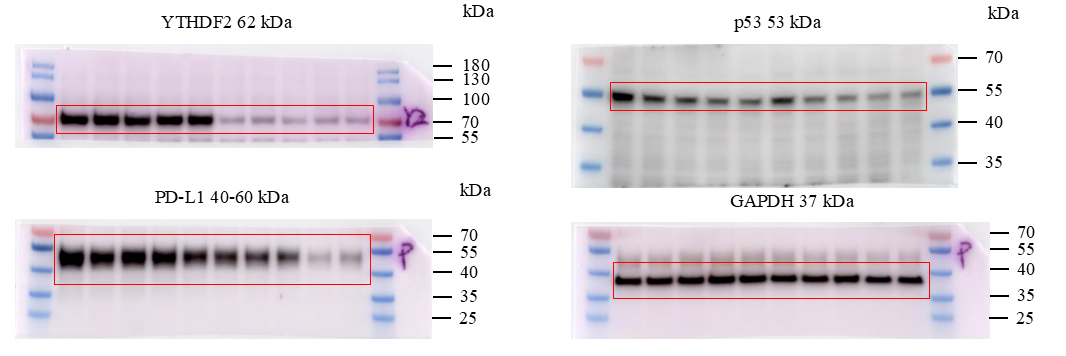


Figure 8 shows the whole blot after cutting membrane at molecular weight 62 kDa, 40-60 kDa, 53 kDa and 37 kDa for YTHDF2 (62 kDa), PD-L1 (40-60 kDa), p53 (53 kDa) and GAPDH (37 kDa).

Figure 9. Original western blots for Figure S5E.


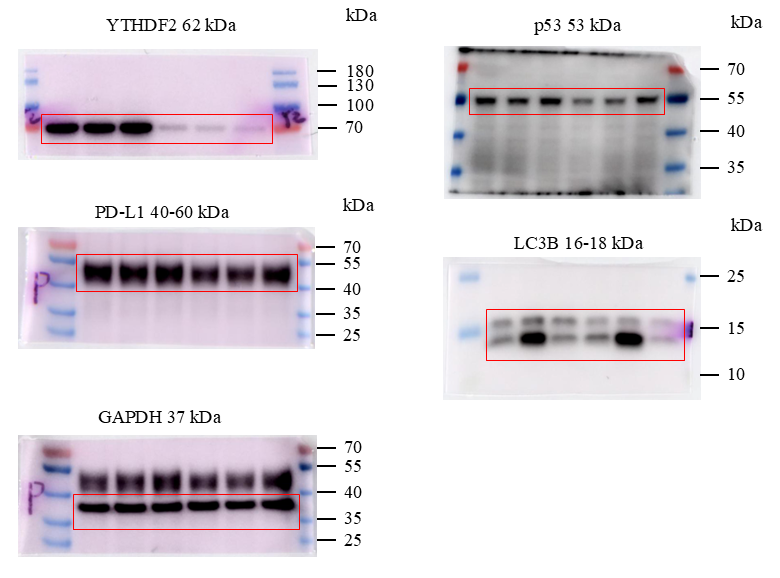


Figure 9 shows the whole blot after cutting membrane at molecular weight 62 kDa, 40-60 kDa, 53 kDa, 16-18 kDa and 37 kDa for YTHDF2 (62 kDa), PD-L1 (40-60 kDa), p53 (53 kDa), LC3B (16-18 kDa) and GAPDH (37 kDa).

Figure 10. Original western blots for Figure S7H.


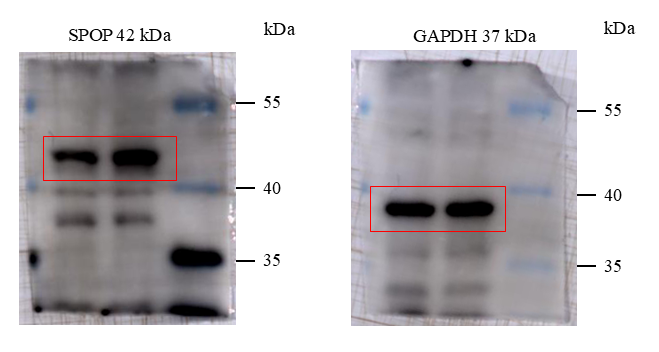


Figure 10 shows the whole blot after cutting membrane at molecular weight 42 kDa and 37 kDa for SPOP (42 kDa) and GAPDH (37 kDa).

Figure 11. Original western blots for Figure S8B.


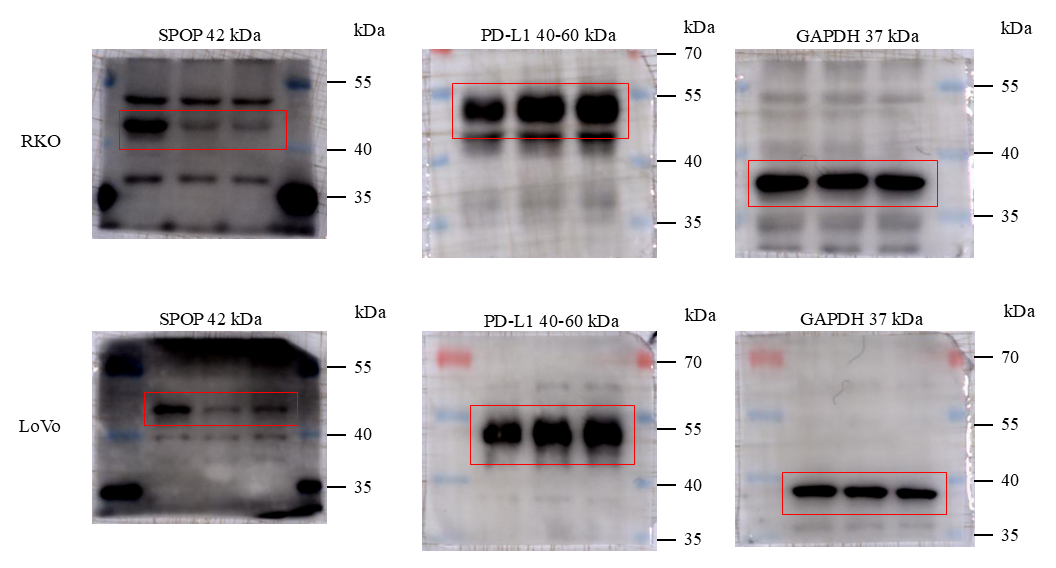


Figure 11 shows the whole blot after cutting membrane at molecular weight 42 kDa, 40-60 kDa and 37 kDa for SPOP (42 kDa), PD-L1 (40-60 kDa) and GAPDH (37 kDa).

Figure 12. Original western blots for Figure S8D.


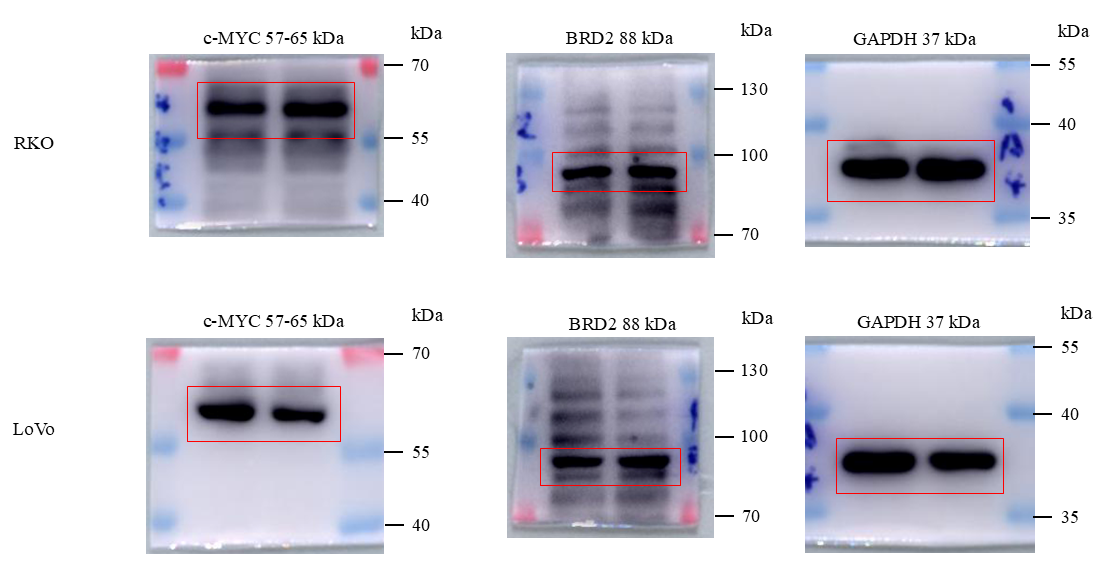


Figure 12 shows the whole blot after cutting membrane at molecular weight 57-65 kDa, 88 kDa and 37 kDa for c-MYC (57-65 kDa), BRD2 (88 kDa) and GAPDH (37 kDa).

Figure 13. Original western blots for Figure S8F.


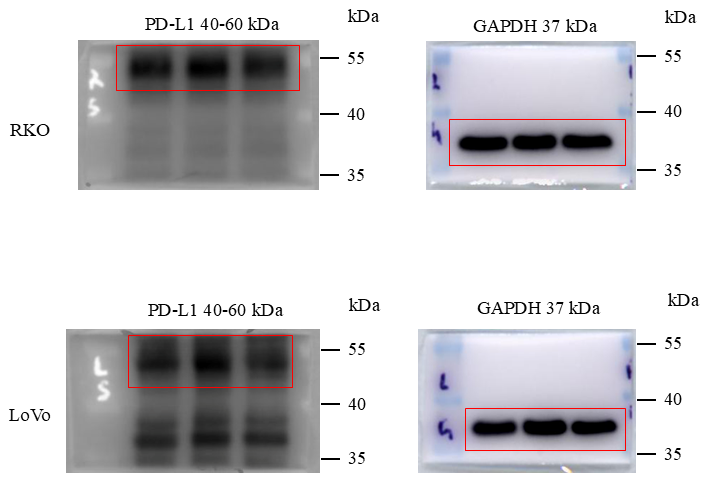


Figure 13 shows the whole blot after cutting membrane at molecular weight 40-60 kDa and 37 kDa for PD-L1 (40-60 kDa) and GAPDH (37 kDa).
